# Structure and Morphology-Controlled Synthesis of Colloidal Ge_1–*x*–*y*_Si_*y*_Sn_*x*_ Quantum Dots with Composition-Tunable Energy Gaps and Visible to Near-IR Optical Properties

**DOI:** 10.1021/acsmaterialsau.5c00164

**Published:** 2025-10-16

**Authors:** Chineme J. Onukwughara, David S. Pate, Yasmitha A. Alahakoon, Ümit Özgür, Indika U. Arachchige

**Affiliations:** † Department of Chemistry, 6889 Virginia Commonwealth University, Richmond, Virginia 23284-2006, United States; ‡ Department of Electrical and Computer Engineering, 6889 Virginia Commonwealth University, Richmond, Virginia 23284-3068, United States

**Keywords:** Group IV semiconductor alloys, quantum dots, direct and indirect energy gaps, visible to near-IR absorption and photoluminescence, core and surface species

## Abstract

Ge_1–*x*–*y*_Si_*y*_Sn_*x*_ quantum dots (QDs) are an attractive class of low-to-nontoxic and earth-abundant semiconductors exhibiting size and composition-tunable optical properties. Their electronic structure can be modified by varying elemental composition and quantum confinement to achieve tunable absorption and photoluminescence (PL) across the visible to near-IR spectrum. Alloying with Sn enhances oscillator strengths, whereas decreasing size and incorporating Si increase energy gaps. Herein, we report a facile colloidal route to produce Ge_1–*x*–*y*_Si_*y*_Sn_*x*_ QDs with narrow size dispersity (4.0 ± 0.4 – 5.2 ± 0.6 nm) and variable Si (*y* = 0.030 – 0.252) and Sn (*x* = 0.044 – 0.059) compositions and investigate the influence of core/surface species on optical properties. Structural analysis reveals an expanded diamond cubic Ge lattice, a red-shifted Ge–Ge Raman peak, and the emergence of a Ge–Si peak with increasing Si composition. Successful alloying of Si and Sn into Ge host lattice is confirmed by electron microscopy, suggesting homogeneous solid solution behavior of ternary QDs. Surface analysis further indicates the presence of Ge^0^/Si^0^/Sn^0^ core species alongside charged Ge^*n*+^/Si^*n*+^/Sn^*n*+^ (1 ≤ *n* ≥ 4) surface species coordinated to passivating organic ligands. The effects of confinement and surface/core elemental composition on optical properties were revealed through composition-tunable absorption onsets (1.15 – 2.33 eV) and associated Tauc direct (1.86 – 3.03 eV) and indirect (1.01 – 1.81 eV) energy gaps achieved for QDs with *x* = 0.044 – 0.059 and *y* = 0.030 – 0.252, which are prominently blue-shifted from bulk counterparts and previously reported Ge_1–*x*_Sn_*x*_ QDs. PL spectra of Ge_1–*x*–*y*_Si_*y*_Sn_*x*_ QDs exhibit nanosecond-scale emission from 1.84 – 1.88 eV for *y* ≤ 0.134 and 2.32 – 2.43 eV for *y* ≥ 0.177 compositions, displaying similarly pronounced blueshifts from comparable Ge_1–*x*_Sn_*x*_ QDs. This correlated absorption/PL tunability expands upon that demonstrated by Ge and Ge_1–*x*_Sn_*x*_ counterparts widens the optical window of Group IV semiconductor nanostructures, making them attractive for visible-to-near-IR optoelectronic studies.

## Graphical abstract

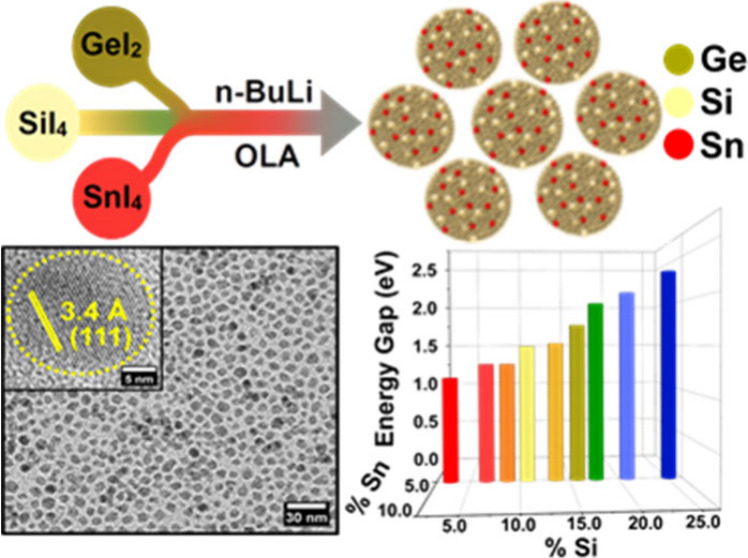

## Introduction

Semiconductor quantum dots (QDs) have, in recent times, demonstrated profound value to society,^1^ emerging as revolutionary materials in modern-day nanotechnology,^2–6^ optoelectronics,^7–9^ sensing,^7–9^ imaging, and energy applications.^7,10^ The driving factor stems from their inherent quantum confinement effects, which lead to size-tunable energy gaps and consequently absorption and photoluminescence (PL) properties. Nevertheless, conventional QDs have typically been centered on Group II–VI and III–V materials (CdX, PbX (X = S, Se, Te), InAs, and GaAs).^3,11–13^ Despite their exceptional optical properties, inherent toxicity of these materials raises significant health and environmental concerns, limiting their widespread use. Therefore, developing novel, environmentally friendly QD systems that are low-to-nontoxic and earth-abundant is essential.

Group IV semiconductors (Si and Ge) offer the advantages of low toxicity, solution processability, and electronic properties that complement the existing Si-based semiconductor technologies.^1,14^ However, bulk Si and Ge possess fundamental indirect bandgaps characterized by low absorption and PL efficiencies, necessitating phonons for optical transitions.^15^ Bandgap engineering can be exploited to overcome this underlying bottleneck and improve light-matter interactions.^16^ Sn alloying enhances the oscillator strengths of electronic transitions, enabling direct gaps in Si_1–*x*_Sn_*x*_ and Ge_1–*x*_Sn_*x*_ bulk alloys, which have gained considerable attention as efficient light emitters.^17–19^ However, this crossover in electronic structure occurs at high Sn compositions (6 – 11%), where a prominent decrease in the Γ-valley compared to the L-valley results in a narrow direct-gap alloy.^10,17,20^ With increasing Sn, the absorption onsets and PL peak energies redshift to longer wavelengths, limiting their applications to mid or far IR spectrum.^21^ Similarly, the significant lattice mismatch between Si vs Sn (∼20%) and Ge vs Sn (∼15%) impedes preserving the structural homogeneity of Group IV alloys. The high Sn content required for an indirect-to-direct-gap crossover increases the probability of Sn segregation; therefore, alloying Si and Ge with Sn has proven extremely challenging.^22^

In recent years, various nonequilibrium growth techniques such as chemical vapor deposition (CVD) and molecular beam epitaxy (MBE) have been utilized to produce Group IV alloys and their thin film nanostructures. Structurally uniform GeSn thin films with Sn content up to ∼34% were fabricated by MBE technique.^23,24^ However, these binary alloys show inferior stability compared to ternary GeSiSn alloys because of the higher entropy and consequently improved stability realized in the latter system.^20,25^ PL maxima spanning from the near to mid-IR spectrum, corresponding to direct gaps of ∼0.49 to 1.00 eV, have been reported for Ge_1–*x*–*y*_Si_*y*_Sn_*x*_ thin films with *x* = 1 – 4% and *y* = 2 – 10% compositions.^21^ For Ge_0.96‑y_Si_0.04_Sn_*y*_ alloys, an indirect to direct-gap crossover has been reported at *y* = ∼9%, at which point the direct-gap (*E*_g_ ≈ 0.5 eV) becomes significantly smaller than the indirect bandgaps of Si (1.1 eV) and Ge (∼0.67 eV).^26^ This energy gap lowering is typically accompanied by increased localized density of states, further signifying the true direct-gap nature of Ge_0.96‑y_Si_0.04_Sn_*y*_ alloys with higher (<9%) Sn compositions.^27^ Moreover, nonthermal plasma synthesis has recently been utilized to produce Si_0.475_Ge_0.475_Sn_0.05_ and Si_0.45_Ge_0.45_Sn_0.10_ nanocrystal (NC) alloys with variable Si/Ge and low (1–2%) Sn compositions.^28^ Here, successful admixing of Si/Ge/Sn has been achieved through secondary precursor injection that kinetically traps Sn in the NC core, yielding larger (10–20 nm), nonquantum confined nanostructures with bulk-like properties. To further extend the absorption and PL energy range, synthesis of low-dimensional nanostructures (QDs,^10,29,30^ nanorods, and wires^31,32^ ) with control over morphology and composition is critically necessary.^30^

Recently, colloidal synthesis methods have been developed to synthesize structurally homogeneous Ge_1–*x*_Sn_*x*_ alloy NCs and QDs with significantly higher Sn content (0 – 95%) than that reported by traditional vapor phase synthesis methods.^8,10,33–41^ Because of their high surface-to-volume ratio and degree of curvature, small colloidal NCs allow structural relaxation that reduces the strain from lattice inhomogeneity, making a wider range of alloy compositions stable. Additionally, the size-tunable confinement energy results in broader tunability of energy gaps across the visible to near-IR spectrum.^42^ For instance, our group reported the synthesis of Ge_1–*x*_Sn_*x*_ QDs with variable diameters (1.8 – 2.3 nm, 3.4 – 4.6 nm, 5.0 – 7.0 nm, and 15 – 23 nm) with Sn content up to ∼30%.^20^ Absorption and PL spectra of 3 – 5 and 5 – 7 nm QDs exhibit strong size confinement effects with composition-tunable onsets and PL maxima in the near IR spectrum.^8,33^ In contrast, ultrasmall (1.8 – 2.2 nm) Ge_1–*x*_Sn_*x*_ QDs exhibit enormous confinement effects and composition-tunable absorption and PL energies in the visible range.^34^ This colloidal strategy has recently been extended to produce bulk GeSiSn alloys (10 – 25 nm) and a few QD compositions.^5^ However, a systematic investigation of the optical properties of QDs as a function of size and composition and the influence of surface vs core species on absorption/PL energies, which are critical for understanding the optical properties of Si/Ge nanostructures, has not been reported. Since Si demonstrates a higher energy gap than Ge and Sn, incorporating Si and decreasing size will widen the spectral range of GeSiSn QDs beyond that reported for GeSn QDs.^43^ Although a few theoretical studies on GeSiSn bulk alloys and matrix-embedded alloy QDs have been reported,^25,44,45^ to our knowledge, the fabrication of Ge_1–*x*–*y*_Si_*y*_Sn_*x*_ QDs as discrete nanostructures and systematic optical investigation as a function of size, composition, surface/core chemical species, and their composition have not been reported.

Herein, we report a facile, low-temperature colloidal route to produce homogeneous Ge_1–*x*–*y*_Si_*y*_Sn_*x*_ alloy QDs with variable Si compositions (*y* = 0.030 – 0.252) while maintaining the Sn composition (*x* = 0.044 – 0.059) and QD diameter (4.0 ± 0.4 to 5.2 ± 0.6 nm) within a narrow range. The Sn composition was maintained near the indirect-to-direct gap transition point (∼6 – 9%) of bulk alloys. The ternary QDs retain the diamond cubic structure of Ge with diffraction patterns shifted to lower 2θ angles, implying homogeneous alloy growth. However, the incorporation of Si in the QD core was lower than that predicted by Vegard’s law.^46–48^ TEM images revealed the pseudospherical morphology and narrow size dispersity of QDs, whereas the elemental maps suggest homogeneous solid solution behavior. Surface analysis of QDs indicates the presence of Ge^0^/Si^0^/Sn^0^ core species and charged Ge^*n*+^/Si^*n*+^/Sn^*n*+^ (1 ≤ *n* ≥ 4) surface species bound to passivating alkylamine and alkane ligands. The alloy QDs exhibit strong size confinement effects, composition-tunable absorption onsets (1.15 – 2.33 eV) and Tauc direct (1.86 – 3.03 eV) and indirect (1.01 – 1.81 eV) energy gaps that are significantly blue–shifted from bulk counterparts for *y* = 0.030 – 0.252 compositions. This broadening of the absorption energy into the visible spectrum is further supported by steady-state and time-resolved PL spectra that show correlated nanosecond–scale emission maxima in distinct regimes: 1.84 – 1.88 eV and 2.32 – 2.43 eV for *y* ≤ 0.134 and *y* ≥ 0.177 compositions, respectively, which remain consistent with varying excitation wavelength. This demonstrates an expansion of absorption/PL energies achievable for Group IV semiconductor QDs and points toward their direct-like core-related nature, making them attractive for a number of new technologies.

## Experimental Methods

### Materials

Germanium­(II) iodide (99.99+%) was purchased from Strem Chemicals. Tin­(IV) iodide (99+%), silicon­(IV) iodide (99%), and 1-octadecene (ODE, 90%) were purchased from Fisher. Oleylamine (OLA, ≥98%), *n*-butyllithium (*n*-BuLi, 1.6 M in hexane), and barium sulfate (BaSO_4_, 99%) were purchased from Sigma-Aldrich. Common solvents such as toluene (≥99.5%), methanol (99+%), chloroform (99.8+%), and carbon tetrachloride (CCl_4_, 99+%) were ACS grade and purchased from Fisher or Acros. Methanol and toluene were dried over molecular sieves and Na, respectively, and distilled under N_2_ prior to use. OLA and ODE were dried at 120 °C under vacuum for 2 h. All other chemicals were used as received. *Caution: Butyllithium compounds are highly pyrophoric and should be handled by properly trained personnel under rigorous air-free conditions.*

### Synthesis of Ge_1–*x*–*y*_Si_*y*_Sn_*x*_ Alloy QDs

Initially, a stock solution of SiI_4_ was freshly prepared by mixing 0.2679 g of SiI_4_ with 10 mL of OLA, followed by heating under vacuum at 120 °C for 90 min. This flask was then cooled to 30 °C, transferred to a glovebox, and stored under N_2_ atmosphere. In a glovebox, appropriate amounts (Table 1) of GeI_2_ and SnI_4_ (0.6 mmol of metal in total) were mixed with 20 mL of OLA in a 50 mL flask and connected to a condenser and thermocouple. This setup was transferred from the glovebox, connected to a Schlenk line, and degassed under 120 °C for 30 min, to produce an orange color solution. Subsequently, the reaction was maintained under N_2_ flow, and the temperature increased to 230 °C. At this point, the appropriate volume of SiI_4_/OLA stock solution (Supporting Information, Table S1) was swiftly injected, causing a temperature drop to ∼215 °C. The red-orange solution was then heated at 210 °C for 20 min, and thereafter, the reaction temperature was raised to 230 °C. Then, an appropriate volume of *n*-BuLi (Supporting Information, Table S1) in 3.0 mL of ODE was swiftly injected, producing a cloudy/smoke-like screen. Subsequently, the reaction was heated to 300 °C and held at this final temperature for 4 min before being cooled with compressed air to ∼60 °C. The synthesis of larger (18.1 – 22.7 nm) Ge_1–*x*–*y*_Si_*y*_Sn_*x*_ alloy NCs followed a similar procedure with the exception that 10 mL of OLA was used and the growth time at 300 °C was increased to 15 min.

**Table 1:** Nominal and Experimental Compositions, Average Particle Diameters, and Absorption Onsets of Ge_1–*x*–*y*_Si_*y*_Sn_*x*_ Alloy QDs with Variable Si Compositions

	Nominal Composition (mmol)				
Experimental Composition^a^	GeI_2_	SiI_4_	SnI_4_	Average Particle Diameter (nm)^b^	Absorption Onset (eV)^c^
Ge_0.698_Si_0.252_Sn_0.050_	0.420	0.132	0.048	4.1 ± 0.3	2.33
Ge_0.745_Si_0.209_Sn_0.046_	0.444	0.108	0.048	4.3 ± 0.5	2.09
Ge_0.779_Si_0.177_Sn_0.044_	0.462	0.090	0.036	4.0 ± 0.4	1.97
Ge_0.785_Si_0.158_Sn_0.057_	0.474	0.078	0.048	4.7 ± 0.5	1.73
Ge_0.810_Si_0.136_Sn_0.054_	0.480	0.072	0.048	4.2 ± 0.6	1.53
Ge_0.832_Si_0.116_Sn_0.052_	0.492	0.060	0.048	5.1 ± 0.6	1.50
Ge_0.845_Si_0.107_Sn_0.048_	0.492	0.060	0.048	4.8 ± 0.7	1.50
Ge_0.855_Si_0.087_Sn_0.058_	0.504	0.042	0.048	4.9 ± 0.7	1.30
Ge_0.879_Si_0.077_Sn_0.044_	0.504	0.042	0.048	4.5 ± 0.6	1.33
Ge_0.888_Si_0.066_Sn_0.046_	0.510	0.048	0.036	4.4 ± 0.5	1.30
Ge_0.907_Si_0.040_Sn_0.053_	0.528	0.030	0.042	4.7 ± 0.6	1.24
Ge_0.911_Si_0.030_Sn_0.059_	0.522	0.030	0.048	5.2 ± 0.6	1.15

a Atomic compositions were obtained from SEM-EDS analysis by averaging five different spots per sample.

b Average QD diameters were calculated from ∼200 particles per sample from multiple LR and HR-TEM images.

c Absorption onsets were estimated by extrapolating the first absorption to the intersection point of the baseline using the least-squares linear regression analysis.

### Isolation and Purification Technique

The as-synthesized QDs were repeatedly washed with toluene and methanol, followed by centrifugation and size-selective precipitation. Upon cooling, the crude particles were transferred to a centrifuge tube. An equivalent amount of methanol was added to the crude solution, followed by centrifugation at 6000 rpm for 10 min to precipitate the QDs. The supernatant was discarded, and 5 mL of toluene was added to the pellet to redisperse the QDs, followed by centrifugation for 5 min. Colloidally stable QDs in toluene were transferred to another tube, and the dissolution/precipitation process was repeated 3 to 4 times to purify QDs. The purified product obtained from the final washing was subjected to size-selective precipitation (SSP) as detailed in the Supporting Information. The size-selected QD fractions were dried under vacuum overnight and stored under N_2_ atmosphere prior to characterization studies.

### Physical Characterization

Powder X-ray diffraction (PXRD) patterns were recorded by using a Malvern PANalytical X-ray diffractometer equipped with Cu Kα (λ = 1.5418 Å) radiation and calibrated with a Si standard. Crystallite sizes were estimated by applying the Scherrer formula to (111) peak of diffraction patterns.^49^ Elemental compositions were investigated by using an energy-dispersive X-ray spectroscopy (EDS) unit attached to a Hitachi SU-70 scanning electron microscope (SEM) operating at 15 kV. A double-sided carbon tape was used to secure the sample onto the SEM holder, and EDS spectra were recorded from 5 individual spots per sample. Low/high-resolution transmission electron microscopy (LRTEM/HRTEM) images, scanning transmission electron microscopy (STEM) and high-angle annular dark field (HAADF) images, and selected area electron diffraction (SAED) patterns were recorded by using a JEM-F200 Cold FEG electron microscope operating at 80 kV. TEM samples were prepared by dispersing QDs in CCl_4_ and drop-casting ∼5 μL solution onto ultrathin carbon-coated copper grids. ImageJ software was used to determine the QD sizes and lattice spacings. A Horiba LABram HR confocal Raman spectrometer was used to record Raman spectra of all samples by using a 532 nm laser. A Cary 6000i UV–visible-near-IR Agilent spectrophotometer equipped with an external diffuse reflectance analysis (DRA) 2500 attachment was used to record the solid-state diffuse reflectance. Powder samples were placed on a BaSO_4_ background holder for reflectance measurements. The Kubelka–Munk remission function was used to convert reflectance data into absorption.^50,51^ Energy gaps were determined by extrapolating from least-squares linear regressions of the first absorption onset of (αhν)^*n*^ to the intersection point of the baseline. Tauc direct and indirect energy gaps were probed by plotting the energy-dependent absorption coefficient F­(R)*hν)^γ^ (obtained from DRA) against photon energy, where the γ factor is equal to 1/2 or 2 for the indirect and direct transitions, respectively.^50,52^ Room-temperature PL spectra of QDs drop-cast on silicon substrates were recorded by utilizing Coherent Obis (405 nm/3.06 eV, ∼10 W/cm^2^) and frequency-doubled Ti:sapphire (for tunable excitation wavelength) lasers in conjunction with a liquid nitrogen-cooled CCD detector mounted onto a 30 cm scanning monochromator for high spectral resolution or a TEC-cooled CCD spectrometer for instantaneous detection of the entire broad spectral range. Corresponding time-resolved data were collected using a PicoQuant 405 nm pulsed laser (∼60 ps fwhm) and Time-Correlated Single Photon Counting (TCSPC) system (<40 ps IRF). Emission spectra were filtered to eliminate scattered excitation light and corrected for the standard response of the measurement systems as obtained via Quartz Tungsten Halogen (QTH) and Hg­(Ar) calibration lamps. X-ray photoelectron spectra (XPS) were recorded using a Thermo Fisher model ESCALAB 250 XPS instrument equipped with Al Kα source (photon energy, 1486.6 eV). Samples were mounted on a molybdenum holder inside a N_2_ glovebox to minimize the oxidation. All spectra were corrected to the C 1s peak (284.8 eV) and analyzed using PHI Multipak software. Fourier-transform infrared (FTIR) spectra of powder samples were recorded using a Thermo Fisher Nicolet iS50 FTIR spectrometer. Thermogravimetric analysis (TGA) was conducted using a TA Instrument Q5000 analyzer under an inert atmosphere. The samples were heated at a constant rate of 50 °C/min.

## Results and Discussion

### Composition and Structural Analysis

In this study, a series of 4 – 6 nm GeSiSn alloy QDs with narrow size dispersity and variable Si compositions was synthesized to systematically investigate the effect of Si composition on energy gaps and absorption/PL properties. The size and Sn composition of QDs were fixed within a narrow range, 4.0 ± 0.4 – 5.2 ± 0.6 nm and 0.044 – 0.059, respectively, while Si composition was varied from 0.030 – 0.252. Control over the QD diameter was achieved by optimizing the reaction parameters, specifically the molar ratio of precursors and reducing agent, growth time, and thorough isolation and purification of QDs with SSP. A lower concentration of precursors than that previously employed for the synthesis of bulk-like GeSiSn alloys was adopted for QD synthesis with a 2-fold increase in OLA to limit particle growth.^4,22,35,42^ SiI_4_/OLA and *n*-BuLi were injected into the reaction below the melting point of β-Sn (231.9 °C)^19^ to ensure the growth of phase-pure alloys by averting undesired impurities.

In a multicomponent system, dynamic variations in reaction parameters such as the concentration of precursors, surfactant ligands, reducing agents, reaction temperature, and growth time can influence the synthesis.^53^ Hence, the stepwise temperature increment and precursor injection method adopted in this study are critical for synthesizing homogeneous alloys. The 20 min heating time at 210 °C following SiI_4_/OLA injection is sufficient to stabilize the precursor mixture, allowing interactions among Si, Ge, and Sn intermediates. The swift injection of BuLi at 230 °C triggers the rapid reduction of halides into the alloy nuclei (Scheme 1). As heating progresses, the solution color changes from reddish-orange to brown-black, signifying a transition from nucleation to particle growth. The time required to ramp the temperature from 230 to 300 °C provides sufficient thermal energy for alloy growth.^54^ A shorter growth time (4 min) at 300 °C proved critical for synthesizing smaller QDs with narrow size dispersity in comparison to longer (≥10 min) growth times used for larger, bulk particles.^4,22^ Rapid quenching with compressed air inhibits the alloy growth, thus, realizing narrowly dispersed QDs with variable Si/Sn compositions. To promote Si incorporation and improved reaction control, a stock solution of SiI_4_ dispersed in OLA was prepared separately prior to incorporation into the GeI_2_/SnI_4_/OLA mixture. This allows the Si precursor to stay in the reactive phase through coordination with OLA, ensuring uniform incorporation into Ge–Sn intermediates.^55–57^ A freshly prepared SiI_4_ stock solution was employed in all syntheses because of the sensitivity of Si to moisture, air, and light. The elemental compositions of Ge_1–*x*–*y*_Si_*y*_Sn_*x*_ QDs were obtained from SEM-EDS and average values are shown in Table 1. Generally, the experimental Sn composition was observed to be ∼3% lower than the nominal moles of SnI_4_ used in the synthesis. On the contrary, the experimental Si composition showed ∼2 – 4% increase from the nominal moles of SiI_4_ used in the synthesis. The atomic compositions of selected QDs were also investigated using XPS (Supporting Information, Table S2), and the values were consistent with those obtained from the SEM-EDS analysis.

**Scheme 1:**
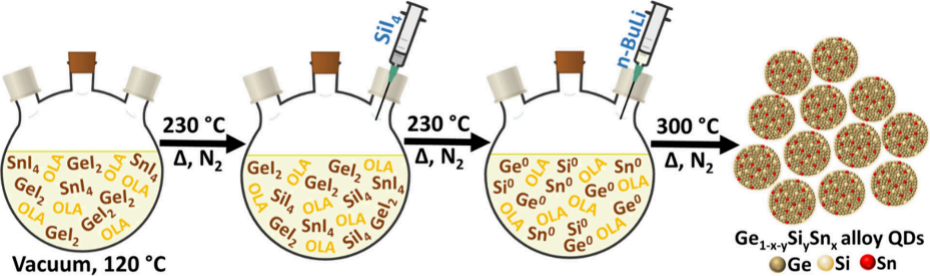
Schematic Representation of the Synthesis of Ge_1–*x*–*y*_Si_*y*_Sn_*x*_ Alloy QDs

Post synthesis, thorough purification cycles involving SSP were adopted to isolate QDs from the crude reaction mixture. This approach not only enabled isolation of smaller QDs by eliminating larger particles and byproducts but also significantly reduced the size dispersity of as-synthesized samples (Supporting Information, Figure S1).^42,58^ In general, the first fraction contains larger polydisperse particles. In the second fraction, the size dispersity began to narrow, and particles were smaller than those in the first fraction. With the third and fourth fractions, uniform QDs with smaller diameters, typically in the range of 4 – 6 nm, were consistently obtained. Given that the last fraction consists of colloidally stable, strongly confined, and narrowly dispersed QDs obtainable for each nominal composition, they were used in all characterization studies. This methodology provides excellent size and size dispersity control of Ge_1–*x*–*y*_Si_*y*_Sn_*x*_ QDs with variable Si compositions, allowing for a systematic study of composition-dependent optical properties.

PXRD patterns and Raman spectra were used to elucidate the structure, crystallinity, and phase purity of the Ge_1–*x*–*y*_Si_*y*_Sn_*x*_ alloys. Diffraction patterns of representative QD compositions are shown in Figure 1A and Supporting Information, Figures S2 – S3. The alloy QDs exhibit Bragg reflections corresponding to the diamond cubic structure of Ge with a noticeable Scherrer broadening, consistent with those reported for Ge and Ge_1–*x*_Sn_*x*_ QDs.^33,36^ Moreover, a moderate decrease in intensity was observed for smaller (4 – 6 nm) QDs as opposed to the intense Bragg reflections observed for larger particles (Supporting Information, Figure S4). Using Scherrer’s equation,^49^ crystallite sizes of ternary QDs were computed from (111) reflection, which showed minor variation (2.4 ± 0.2 – 3.0 ± 0.3 nm) across variable Si compositions (*y* = 0.030 – 0.252). This narrow variation can be attributed to the relatively consistent Sn compositions (*x* = 0.044 – 0.059) achieved during the synthesis. Furthermore, diffraction patterns were shifted to lower 2θ angles compared to the Ge reference pattern. This shift can be attributed to the expansion of the diamond cubic Ge structure by α-Sn.^30^ In general, minor shifts toward higher 2θ angles were observed at low to intermediate Si compositions (*y* = 0.030 – 0.177), while more pronounced shifts were observed at higher Si (*y* = 0.209 and 0.252) compositions (Supporting Information, Figure S3). The dominant Sn-induced lattice expansion is believed to outweigh the lattice contraction expected from Si incorporation, resulting in minimal peak shifts, specifically at lower Si compositions. Additionally, the lattice parameters were computed using (111) peaks of PXRD patterns following pseudo-Voigt fits, as well as using Vegard’s law^46,47^ and quadratic parameters reported by Moontragoon et al.^48^ (Supporting Information, Figure S5). The comparatively larger lattice parameters obtained for QDs compared with those expected at the respective EDS compositions suggest low Si incorporation in the QD core, which is further supported by XPS and PL analyses discussed below. In contrast, the lattice constants computed using the parameters reported by Moontragoon et al. and Vegard’s law illustrate the expected near-linear decrease with increasing Si composition in this ternary alloy system.

**Figure 1:**
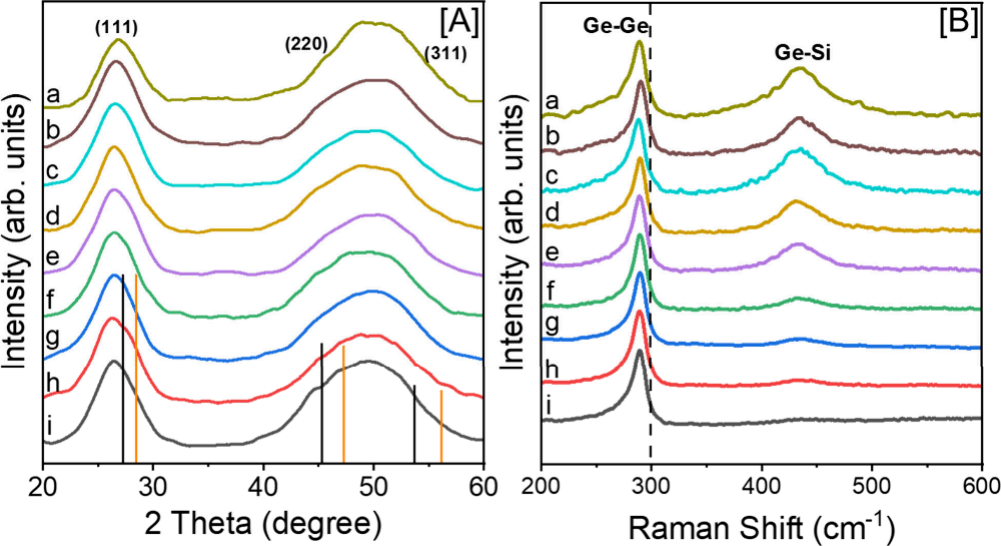
[A] PXRD patterns and [B] Raman spectra of alloy QDs with variable elemental compositions: (a) Ge_0.698_Si_0.252_Sn_0.050_, (b) Ge_0.745_Si_0.209_Sn_0.046_, (c) Ge_0.779_Si_0.177_Sn_0.044_, (d) Ge_0.810_Si_0.136_Sn_0.054_, (e) Ge_0.832_Si_0.116_Sn_0.052_, (f) Ge_0.855_Si_0.087_Sn_0.058_, (g) Ge_0.879_Si_0.077_Sn_0.044_, (h) Ge_0.888_Si_0.066_Sn_0.046_, and (i) Ge_0.911_Si_0.030_Sn_0.059_. The ICCD-PDF overlays of diamond cubic Ge (ICCD no. 04–002–0892) and Si (ICCD no. 01–085–8586) are shown in vertical black and orange lines, respectively. The dashed line in [B] represents the bulk Ge–Ge vibration at 300 cm^–1^.

Raman spectra of as-synthesized Ge_1–*x*–*y*_Si_*y*_Sn_*x*_ QDs were used to investigate the effects of Si/Sn alloying on local bonds (Figure 1B and Supporting Information, Figure S2 – S3). For bulk Ge, a Raman peak corresponding to Ge–Ge bond is expected at 300 cm^–1^,^31,59^ whereas for Ge QDs, a red-shifted peak is expected at 297 – 300 cm^–1^ owing to phonon confinement.^60^ Similarly, a red-shifted Ge–Ge peak has been reported for Ge_1–*x*_Sn_*x*_ QDs (295 – 287 cm^–1^) owing to Sn alloying.^8,33^ For Ge_1–*x*–*y*_Si_*y*_Sn_*x*_ alloy QDs, two distinct Raman peaks were observed; an intense Ge–Ge peak with an asymmetrical shoulder appearing at ∼289 – 291 cm^–1^ along with a broad Ge–Si peak appearing at ∼431 – 437 cm^–1^_,_ which becomes more prominent when Si content is >0.066. The red-shifting and asymmetry of the Ge–Ge peak can be attributed to the combined effects of Sn alloying and phonon confinement inherent in smaller QDs.^47,61^ The emergence of Ge–Si peak is indicative of the successful admixing of Si into the Ge host lattice (Supporting Information, Table S3).^62^ These observations reflect the modulation of lattice vibrations due to Si/Sn incorporation because of the differing masses and atomic radii of Si, Ge, and Sn.

Low-resolution TEM images were recorded to investigate the morphology and size dispersity of Ge_1–*x*–*y*_Si_*y*_Sn_*x*_ alloy QDs (Figure 2). For all compositions, QDs appear as discrete particles with a quasi-spherical morphology and narrow size dispersity. The average size of Ge_1–y–x_Si_*y*_Sn_*x*_ QDs varies from 4.0 ± 0.4 – 5.2 ± 0.6 nm for *y* = 0.030 – 0.252 compositions. Additional LRTEM images and size histograms are shown in the Supporting Information, Figure S6 – S9. A low voltage (80 kV) TEM imaging approach was used to overcome the minimal Z-contrast between Si and the carbon support, enabling more precise size measurements. Additionally, thorough purification and SSP were used to eliminate organic impurities, which contributed to image obscurity.^3,63^ The polydispersity index of QDs showed values <0.1, suggesting the narrow size dispersity of all compositions (Supporting Information, Table S3).^64,65^ When the standard deviation is expressed as a percentage of the mean size for each composition, size dispersity ranges from 7.3 – 14.6%. Moreover, Si and Sn compositions significantly impacted the average QD diameter. For instance, QDs with the highest Si and lowest Sn content (Ge_0.698_Si_0.252_Sn_0.050_, Ge_0.745_Si_0.209_Sn_0.046_, and Ge_0.779_Si_0.177_Sn_0.044_) displayed the smallest particle size. In contrast, Ge_0.911_Si_0.030_Sn_0.059_ QDs with the lowest Si and highest Sn content showed the largest particle size (Supporting Information, Figure S6 and S9). Sn with a larger atomic radius than Si and Ge promotes larger particle growth, which has also been observed with the Ge_1–*x*_Sn_*x*_ QD system. In contrast, higher Si favors smaller, more narrowly dispersed alloy growth.^33^ Lattice fringes obtained from Ge_0.832_Si_0.116_Sn_0.052_ and Ge_0.907_Si_0.040_Sn_0.053_ QDs (Supporting Information, Figure S10) exhibit d_(111)_-spacing of 3.30 Å, and 3.34 Å, respectively which are larger than that of Ge QDs (3.27 Å),^66^ indicating an expansion of diamond cubic Ge structure owing to Sn incorporation.

**Figure 2:**
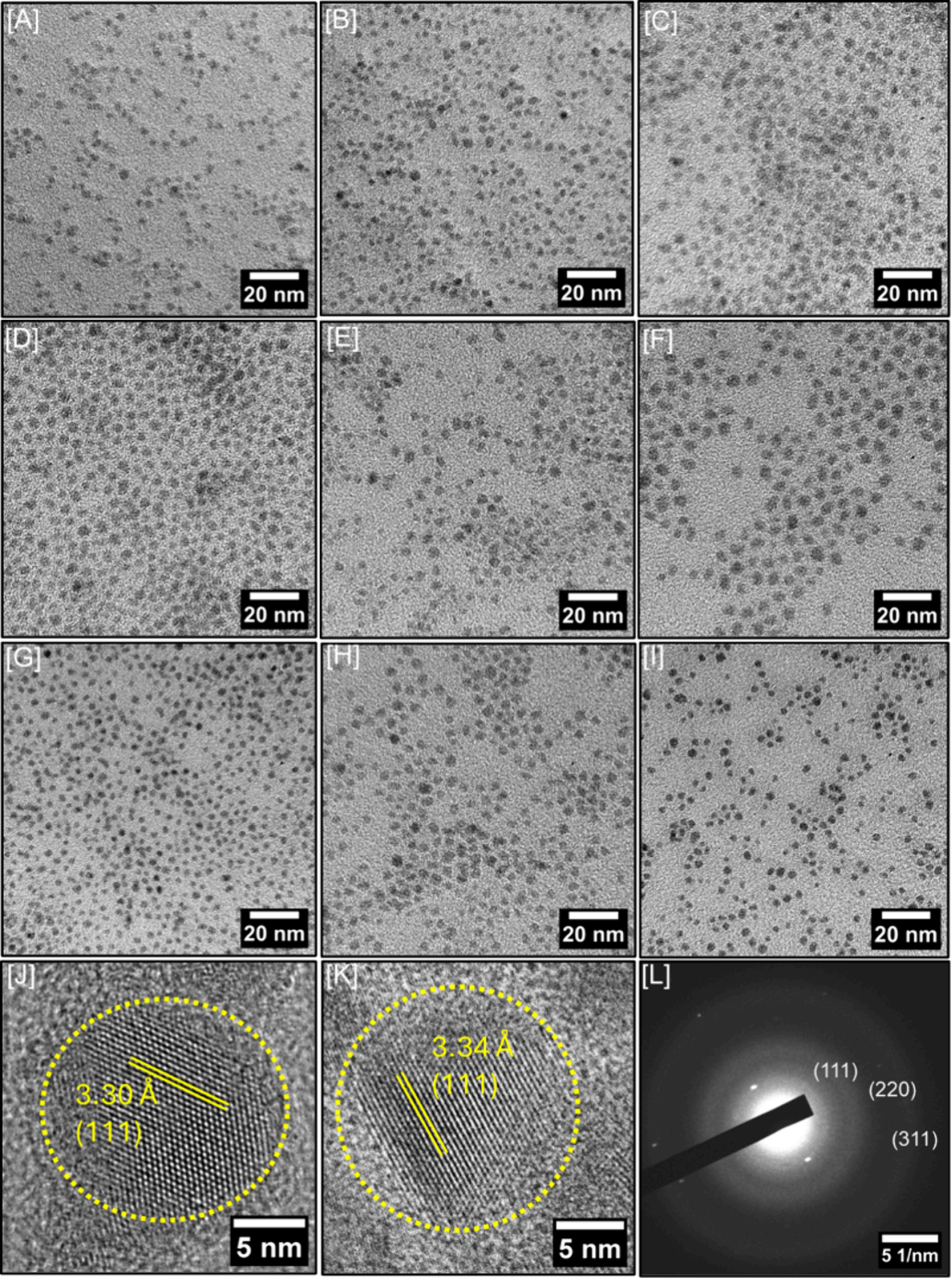
Low-resolution TEM images of Ge_1–*x*–*y*_Si_*y*_Sn_*x*_ alloy QDs with variable compositions: [A] Ge_0.698_Si_0.252_Sn_0.050_, [B] Ge_0.745_Si_0.209_Sn_0.046_, [C] Ge_0.779_Si_0.177_Sn_0.044_, [D] Ge_0.785_Si_0.158_Sn_0.057_, [E] Ge_0.810_Si_0.136_Sn_0.054_, [F] Ge_0.832_Si_0.116_Sn_0.052_, [G] Ge_0.855_Si_0.087_Sn_0.058_, [H] Ge_0.888_Si_0.066_Sn_0.046_, and [I] Ge_0.907_Si_0.040_Sn_0.053_. HRTEM images of [J] Ge_0.832_Si_0.116_Sn_0.052_ and [K] Ge_0.907_Si_0.040_Sn_0.053_ QDs display an expanded (111) lattice spacing. [L] A representative SAED pattern of Ge_0.832_Si_0.116_Sn_0.052_ QDs. Lattice spacings were calculated using ImageJ software by processing the inverse FFT of corresponding HRTEM images.^37^

To investigate the structural homogeneity of alloy QDs, STEM-HAADF images were recorded (Figure 3 and Supporting Information, Figure S11). Smaller particles, when subjected to an intense electron beam, often degrade during STEM imaging. Therefore, a polydisperse mixture of larger, nonquantum-confined alloys was produced for STEM analysis. The elemental maps recorded from larger alloys indicate uniform Ge, Si, and Sn distribution throughout discrete particles, suggesting homogeneous solid solution behavior. A similar elemental distribution is expected for both smaller and larger alloys since smaller QDs act as seeds for larger particle growth, consistent with the LaMer model^67^ of colloidal NC growth.^8,34,37,68^ This further supports the synthesis of phase-pure, diamond cubic Ge_1–*x*–*y*_Si_*y*_Sn_*x*_ alloys without impurities emanating from phase segregation. However, note that these maps represent integration throughout the entire depth of the sample area and therefore cannot distinguish between surface and core elemental positions or confirm volumetric homogeneity.

**Figure 3:**
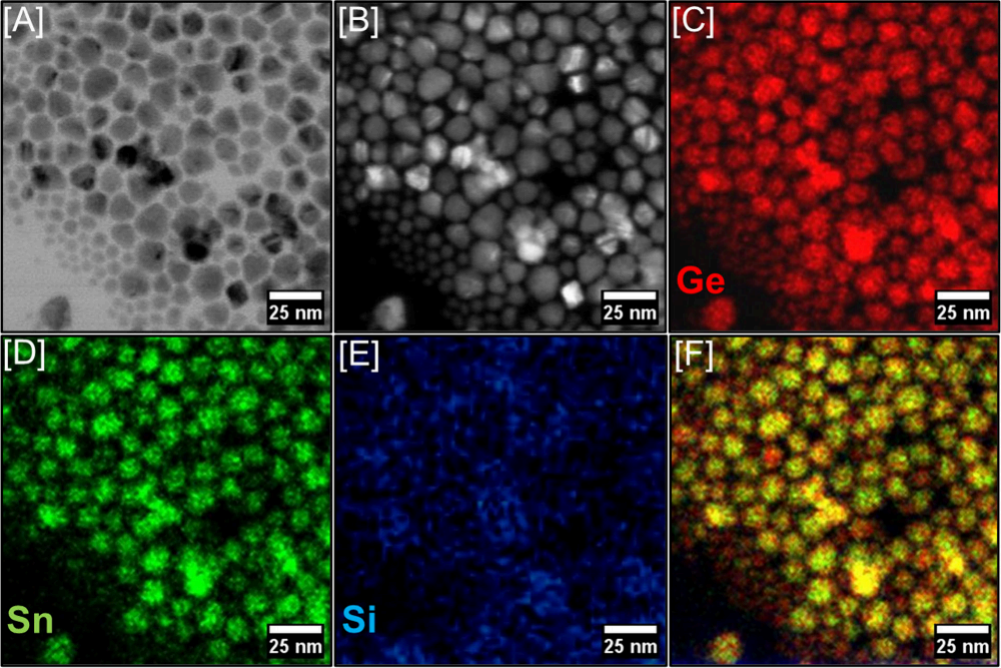
[A] Bright-field and [B] dark-field TEM images of Ge_0.831_Si_0.065_Sn_0.104_ alloys along with STEM-HAADF elemental maps of [C] Ge, [D] Sn, [E] Si, and [F] overlays of all elements, suggesting the homogeneous solid solution behavior of as-synthesized alloys.

To differentiate surface and core oxidation states, chemical species, and passivating surface ligands, alloy QDs were characterized by XPS and FTIR spectroscopy. Representative Ge 3d, Si 2p, and Sn 3d XPS spectra of Ge_0.810_Si_0.136_Sn_0.054_ QDs are shown in Figure 4A-C. The Ge 3d spectrum (Figure 4A) exhibits two distinct peaks centered at 29.3 and 32.2 eV that can be attributed to the Ge^0^ core and Ge^*n*+^ surface species, respectively. The deconvolution of the low energy peak produces two peaks at 29.2 and 29.9 eV, which can be attributed to the Ge^0^_5/2_ and Ge^0^_3/2_ core species.^69^ The deconvoluted peaks observed at 31.4 and 32.4 eV can be assigned to Ge^2+^ and Ge^2+^/Ge^3+^ surface species, originating from multimodal passivation of surface Ge atoms by surfactant ligands.^18^ These peaks are consistent with previous findings in Ge-based nanostructures.^33,70^ The integrated area under the Ge^0^ XPS peaks constitutes 74.3 – 76.8% of the total integrated area for all Ge species (Supporting Information, Figure S12, Tables S2 and S4). This is indicative of the percentile of Ge atoms in the core and is consistent with that expected from a spherical QD of diameter 4.2 nm (80%). The Si 2p spectrum (Figure 4B) exhibits a deconvoluted peak at 99.3 eV corresponding to Si^0^ core species.^71,72^ The dominant peak, centered at 102.3 eV can be attributed to charged surface Si states (Si^1+^/Si^2+^/Si-X, X = C or N), stabilized by surface ligand interactions.^71,73,74^ Additionally, the high energy deconvoluted peak observed at 104.2 eV corresponds to Si^4+^ species, originating from surface oxide species formed during handling of QDs in the ambient atmosphere.^75,76^ Overall, we observed a lower contribution from the Si^0^ peak compared to Ge^0^ and Sn^0^, compared to that indicated by EDS, as evidenced by the smaller ratio of the area under the Si^0^ peak (1.3 – 10.8%) to the total integrated area for all Si species (Supporting Information, Figure S12 and Table S4). This further supports a decrease in the number of core-incorporated Si atoms (Table S2), consistent with lattice parameters computed from PXRD studies (Supporting Information, Figure S5). In the Sn 3d spectrum (Figure 4C), deconvoluted peaks at 484.9 and 493.2 eV can be attributed to core Sn^0^ species originating from 3d_5/2_ and 3d_3/2_ spin–orbit coupling, respectively, consistent with the literature.^35^ The higher energy deconvoluted peaks observed at 486.5 and 494.9 eV can be assigned to charge surface species (Sn^2+^/Sn^4+^) bound to surfactant ligands.^70^ The integrated areas under Sn^0^ peaks constitute 33.6 – 64.7% of the total area, also suggesting a slight decrease in the Sn^0^ atoms incorporated in the QD core (Supporting Information, Figure S12, Tables S2 and S4). Overall, XPS spectra reveal that alloy QDs consist of a core structure where Ge/Si/Sn species are present in their zerovalent states, along with charged surface species bound to passivating organic ligands (OLA, ODE, or butyl).

**Figure 4:**
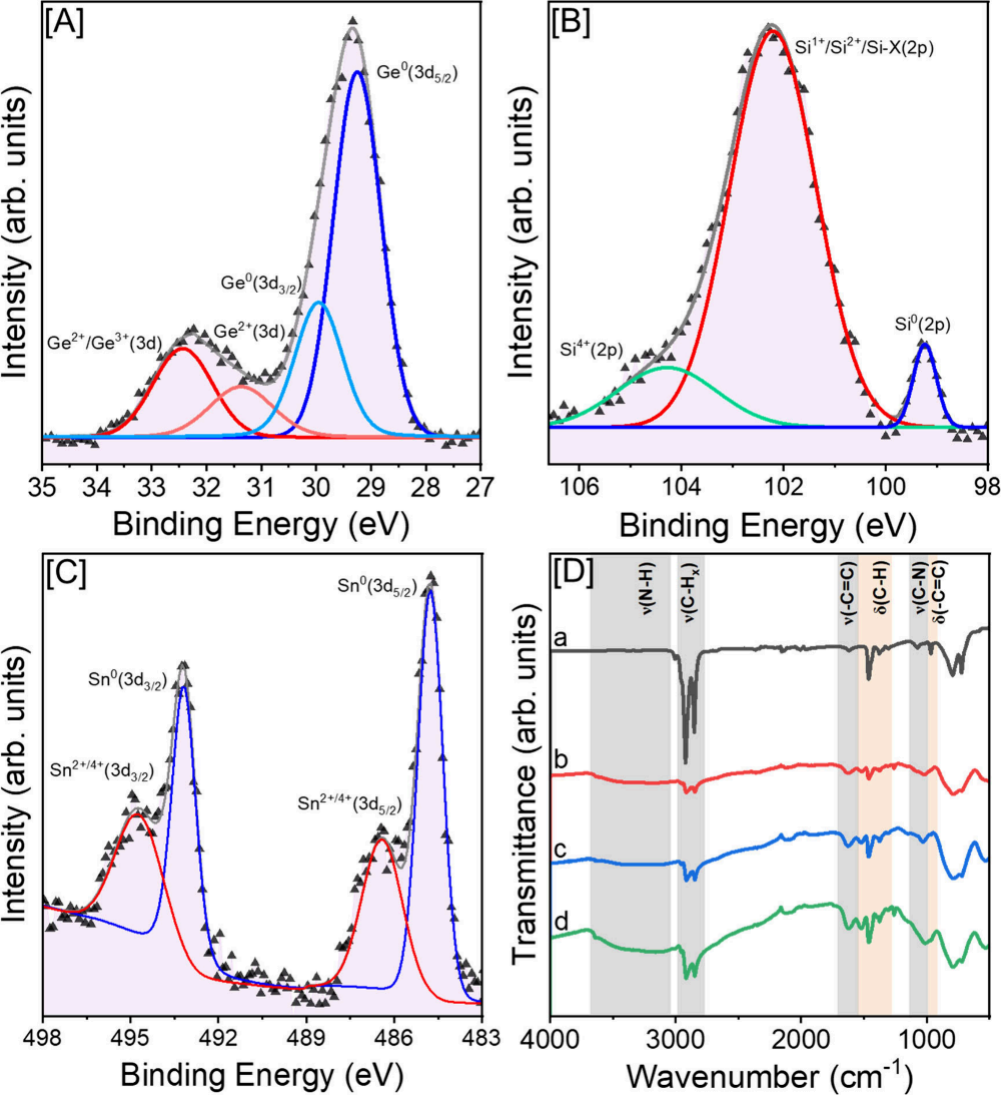
[A] Ge 3d, [B] Si 2p, and [C] Sn 3d XPS spectra of Ge_0.810_Si_0.136_Sn_0.054_ alloy QDs. The black symbols represent experimental data; blue and red lines are deconvolution peaks fitted to Ge^0^/Si^0^/Sn^0^ species and different oxidation states of Ge^*n*+^/Si^*n*+^/Sn^*n*+^ species, respectively, and gray lines are spectral envelopes. [D] FTIR spectra of (a) OLA along with (b) Ge_0.911_Si_0.030_Sn_0.059_, (c) Ge_0.861_Si_0.088_Sn_0.052_, and (d) Ge_0.810_Si_0.136_Sn_0.054_ alloy QDs.

The FTIR spectra of Ge_0.911_Si_0.030_Sn_0.059_, Ge_0.861_Si_0.088_Sn_0.052_, and Ge_0.810_Si_0.136_Sn_0.054_ QDs were recorded to further investigate the surface characteristics of ternary alloys (Figure 4D). Sharp peaks corresponding to the CH_*x*_ stretching (2850 – 2958 cm^–1^) and bending (1465 – 1292 cm^–1^) vibrations were evident in all QDs, suggesting the presence of hydrocarbons associated with the surface ligands (OLA and ODE).^71^ The broad peak at ∼1624 cm^–1^ can be assigned to -C=C- stretching vibrations, originating from the unsaturated oleyl chain.^77^ Additionally, the presence of N–H stretching (∼3044 – 3590 cm^–1^) and bending (∼1520 cm^–1^) vibrations emanating from the primary amine group further supports the surface passivation by OLA ligands. The broadening observed beyond 3000 cm^–1^, not visible in free OLA, suggests notable ligand–QD binding interactions, consistent with effective surface passivation of QDs by the OLA ligands. Subtle variations in intensities observed in the above peaks across different QD compositions indicate differences in ligand-QD interactions, likely influenced by the variation in surface species/composition of QDs, consistent with XPS studies. Overall, characteristic vibrations observed for surface-bound alkyl and alkylamine groups, along with the presence of charged Si^n+/^Ge^n+/^Sn^*n*+^ species from XPS, confirm the successful passivation of alloy QDs with organic ligands, accounting for their colloidal stability in nonpolar solvents. This is also supported by TGA analysis (Supporting Information, Figure S13), which shows the complete removal of passivating surface ligands upon annealing under an inert atmosphere.

### Optical Analysis

Solid-state diffuse reflectance spectroscopy was employed to probe the absorption onsets and energy gaps of Ge_1–*x*–*y*_Si_*y*_Sn_*x*_ QDs as a function of the Si composition. The reflectance data were converted to absorption using the Kubelka–Munk (KM) remission function,^50^ and the onsets were estimated from extrapolation of the first absorption to the intersection point of the baseline using the least-squares linear regression analysis (Supporting Information, Figure S14). The Ge_1–*x*–*y*_Si_*y*_Sn_*x*_ alloy QDs exhibit well-defined onsets from 1.15 – 2.33 eV, (Figure 5A and Supporting Information, Figure S15 – 16 and Table S5), showing a systematic blueshift with increasing Si (*y* = 0.030 – 0.252) for a near-consistent QD size (4.0 ± 0.4 – 5.2 ± 0.6 nm) and Sn composition (*x* = 0.044 – 0.059), suggesting that alloying with Si widens the fundamental energy gaps. These onsets denote substantial blueshifts from those reported for bulk Ge (0.66 eV) and Ge_1–*x*_Sn_*x*_ alloy QDs (1.08 – 1.22 eV^33^ for 4.0 ± 0.8 – 4.6 ± 1.2 nm and 0.84 – 1.22 eV^8^ for 4.4 ± 0.7 – 5.0 ± 0.7 nm, respectively) with comparable size and Sn compositions. Similarly, the energy gaps estimated for Ge_1–*x*–*y*_Si_*y*_Sn_*x*_ QDs are significantly larger than those reported for Ge_1–*x*–*y*_Si_*x*_Sn_*y*_ alloy thin films (0.9 – 1.2 eV, for *x* = 0.070 – 0.100 and *y* = 0.020 – 0.110),^62,78^ consistent with the quantum confinement effects. Reflecting on the direct influence of Si, Ge_0.698_Si_0.252_Sn_0.050_ and Ge_0.913_Si_0.030_Sn_0.059_ QDs with the highest and lowest Si compositions, respectively, display the highest and lowest energy gaps (2.33 and 1.15 eV) among all samples. However, subtle variations in size and Sn content can offset the influence of Si on the energy gaps. For instance, despite having a higher Si content, Ge_0.855_Si_0.087_Sn_0.058_ QDs exhibit a slightly lower absorption onset (1.30 eV) compared to Ge_0.879_Si_0.077_Sn_0.044_ QDs (1.33 eV) with a lower Si content (Table 1). This discrepancy can be attributed to minor variations in QD size (4.9 ± 0.7 nm vs 4.5 ± 0.6 nm) and/or Sn composition (0.058 vs 0.044). These trends highlight the intricate interplay between size and composition in defining the energy gaps of ternary QDs.^42^

**Figure 5:**
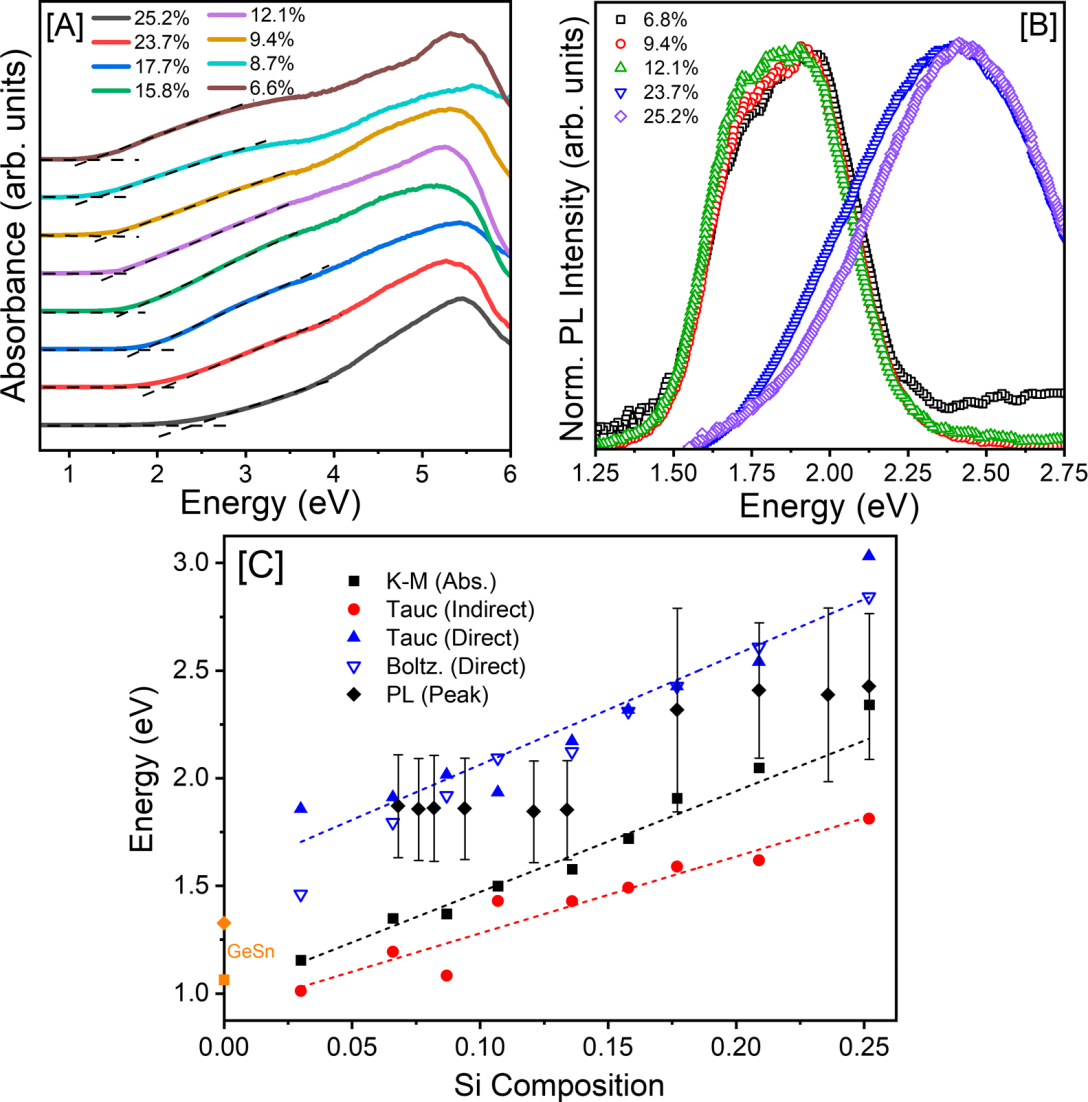
[A] Solid-state absorption spectra of selected Ge_1–*x*–*y*_Si_*y*_Sn_*x*_ alloy QDs with different Si compositions. Additional absorption data of all QD compositions are shown in Supporting Information Table S5. [B] Representative PL spectra obtained for Ge_1–*x*–*y*_Si_*y*_Sn_*x*_ alloy QDs with variable Si compositions, *y* = 0.068 – 0.252. [C] Energy gaps of Ge_1–*x*–*y*_Si_*y*_Sn_*x*_ QDs with variable Si composition obtained via PL (black diamonds, peak with fwhm indicated as error bars) and multiple absorption processing methods, Kubelka–Munk (black squares), indirect Tauc (red circles), direct Tauc (filled blue triangles), and direct Boltzmann Sigmoid (hollow blue triangles) analyses. An orange square and diamond representing average absorption and PL peak energies from comparable GeSn QDs (4.4 – 4.6 nm having zero Si and 0.056 – 0.079 Sn content) are shown on the energy axis for comparison.^8^

Optical properties of alloy QDs were further explored by additional analysis of solid-state absorption spectra (Figure 5C and Supporting Information, Table S5). Although potentially limited in its applicability for such nanostructures, Tauc analysis of several distinct absorption contributions provides additional insight into the residual bulk-like nature and the behavior of indirect and direct-like transitions. For example, KM absorption onsets align closely to the Tauc indirect gap transitions (1.01 – 1.81 eV) across the Si composition range, likely corresponding to transitions of potentially mixed character primarily involving the Ge-rich QD core. In contrast, the direct Tauc curves revealed a strong direct absorption component ranging from 1.85 – 3.03 eV and increasing with Si content (Figure 5C). Following analogous trends, additional confirmatory Boltzmann Sigmoidal fitting^79^ to the absorption indicated similar values for the fundamental direct transition (1.46 – 2.84 eV). The consistent increase in energy gaps with increasing Si composition across all analyses further illustrates the influence of Si on widening the energy gaps of ternary QDs.

In conjunction with absorption, steady-state PL spectra were obtained for a representative collection of the Ge_1–*x*–*y*_Si_*y*_Sn_*x*_ QDs, shown in Figure 5B and Supporting Information, Table S6. Appearing in distinct regimes, emission between 1.84 and 1.88 eV with an average fwhm of 0.47 eV was observed for QDs having Si content ≤ 0.134 as relatively weak PL peaks compared to the wider, strong PL peaks ranging 2.32 – 2.43 eV (average fwhm of 0.76 eV) measured in samples with higher Si content (0.177 – 0.252). The slight peak asymmetries are likely a result of compositional or size polydispersity variations within the measured QD sample area. Although near the direct energy gaps obtained via Tauc and Boltzmann analyses (significantly blue-shifted compared to the indicated KM and indirect Tauc energy gaps), the origins, distinctions, and true indirect/direct nature of such separated PL peaks are so far unclear. As clearly evident in Figures 5C and S17 in the Supporting Information, significant PL blueshifts and faster recombination decays (0.41 – 3.39 ns) compared to Ge_1–*x*_Sn_*x*_ QDs of similar size and composition (∼10 ns)^8,10^ indicate the influence of Si incorporation in the QD core.^80^ However, there is an apparent reduction in the PL tunability of the Ge_1–*x*–*y*_Si_*y*_Sn_*x*_ QDs with respect to the generally less susceptible optical absorption. This can likely be attributed to a reduced core Si^0^ concentration (up to only ∼0.05 suggested by XRD and XPS analyses as summarized in Table S2) local to the emission progenitor compared to the average values measured by EDS (up to *y* = 0.252), as discussed above. As shown in Supporting Information, Figure S17, fast room-temperature time-resolved PL transients were observed from the QDs at both low and high Si incorporation regimes. These decays displayed distinct dependence on Si composition, ranging from τ_Av_ = 0.41 ns for *y* = 0.082 to τ_Av_ = 3.39 ns for *y* = 0.209. Luminescent surface or mixed surface-core defects^81,82^ can also arise from residual charged Si species, which may additionally contribute to the observed ns-scale emission. However, the absence of a notable emission shift with excitation wavelength (Supporting Information, Figure S18), alongside consistent nanosecond-scale decays, suggests the observed direct-like emission is primarily core related. Specific studies to investigate multiple radiative decay pathways are currently underway and will be published elsewhere.

## Conclusions

We have successfully developed a low-temperature colloidal synthetic route to produce Ge_1–*x*–*y*_Si_*y*_Sn_*x*_ alloy QDs with a fixed Sn composition (0.044 – 0.059) and variable Si composition (0.030 – 0.252) while maintaining the average diameter within a narrow range (4.0 ± 0.4 nm – 5.2 ± 0.6 nm). The homogeneous alloying of Ge/Si/Sn was confirmed through the PXRD patterns and STEM-EDS elemental maps. TEM analysis indicates the quasi-spherical morphology and narrow size dispersity of QDs with variable Si compositions. The alloy QDs exhibit an expanded diamond cubic Ge structure, resulting from the admixing of Si and Sn. However, the Si composition in the QD core was lower than that indicated by the EDS studies, which is further supported by XPS and PL analyses. Two prominent Raman peaks were observed: a red-shifted Ge–Ge peak at ∼289 – 291 cm^–1^, attributed to the combined effects of phonon confinement and Sn alloying, and a Ge–Si peak at ∼431 – 437 cm^–1^, which becomes increasingly stronger with increasing Si composition. Surface analysis confirmed the presence of zerovalent and charged states attributed to core Ge^0^/Si^0^/Sn^0^ and surface Ge^*n*+^/Si^*n*+^/Sn^*n*+^ species bonded with passivating surface ligands. As-synthesized alloys demonstrate strong size confinement effects with systematic blueshifts in absorption onsets (1.15 eV – 2.33 eV) and Tauc direct (1.86 – 3.03 eV) and indirect (1.01 – 1.81 eV) energy gaps with increasing Si composition (*y* = 0.030 – 0.252). These onsets are significantly higher than those reported for bulk Ge and GeSn QDs, highlighting the influence of Si incorporation. PL spectra show fast direct-like low and high energy emission centered at 1.84 – 1.88 eV and 2.32 – 2.43 eV for QDs with *y* ≤ 0.134 and *y* ≥ 0.177, respectively, further illustrating the correlated effects of core and surface Si incorporation on optical properties. Combined, these results provide a foundation for further studies into this promising ternary QD system and probing of their potential in optoelectronic studies. Specific studies to test these premises are currently underway.

## Supplementary Material



## Notes

Procedure for size-selective precipitation, experimental and nominal compositions of alloy QDs, Raman peak positions, average particle sizes and size dispersity data; TEM images of QD fractions, zoomed in PXRD patterns and Raman spectra, PXRD plot for bulk-like particles, comparative plot of theoretical and experimental lattice parameters, additional LRTEM images of ternary QDs along with size histograms, additional XPS spectra, comparison of XPS and SEM-EDS composition, TGA curves of free OLA and OLA passivated QDs, representative fits of solid state absorption spectra, energy gap data obtained from Kubelka–Munk, Tauc direct- and indirect-gap equations, Boltzmann analyses of QDs, PL peak energy values of QDs, TRPL decays for selected QDs, excitation wavelength dependent PL, and LRTEM, HRTEM, and STEM-EDS maps of larger alloy NCs (PDF)
