# Supplementary material for: Structure and Morphology-Controlled Synthesis of Colloidal Ge1–x–y Si y Sn x Quantum Dots with Composition-Tunable Energy Gaps and Visible to Near-IR Optical Properties
Source: ACS Mater Au. 2025 Oct 16;5(6):1080–91. doi: 10.1021/acsmaterialsau.5c00164 (PMC12616438; doi:10.1021/acsmaterialsau.5c00164)
Supplement: Supplementary file 1 [file mg5c00164_si_001.pdf]

Supporting Information

Structure and Morphology-Controlled Synthesis of Colloidal $Ge_{1-x-y}Si_ySn_x$ Quantum Dots with Composition-Tunable Energy Gaps and Visible to Near-IR Optical Properties

*Chineme J. Onukwughara,[1,§] David S. Pate,[2,§] Yasmitha A. Alahakoon,[1] Ümit Özgür,[2] and Indika U. Arachchige[1,]**

[1]Department of Chemistry, Virginia Commonwealth University, Richmond, Virginia 23284-2006, USA

[2]Department of Electrical and Computer Engineering, Virginia Commonwealth University, Richmond, Virginia 23284-3068, USA

[§]C.J.O. and D.S.P. contributed equally to this work

*Email: iuarachchige@vcu.edu

Procedure for Size Selective Precipitation

The QD precipitate obtained from isolation and purification was subjected to size-selective precipitation, as discussed below. This precipitate was redispersed in 5 mL of toluene, producing a dark-brown solution. To this solution, 45 drops of methanol were added using a transfer pipette at the rate of 1 mL/min. The solution was simultaneously swirled to ensure adequate mixing, followed by centrifugation for 10 min at 6000 rpm to obtain the first QD fraction. The supernatant was then transferred to another tube, where 30 drops of additional methanol were added dropwise, followed by centrifugation to obtain the second fraction, after which the supernatant was transferred to another tube. This process was repeated until the complete precipitation of QDs using 25 to 30 drops of methanol to obtain the third and fourth fractions, respectively. At this stage, the supernatant became clear, indicating the successful precipitation of all QDs, and was subsequently discarded. The size-selected QD fractions were vacuum-dried and stored under N_2.

Table S1. Experimental QD composition, nominal volume of 0.05 M SiI_4 solution, and millimoles of n-BuLi used in the synthesis of $Ge_{1-x-y}Si_ySn_x$ alloy QDs.

QD Composition	BuLi (mmol)	Volume of 0.05 M SiI_4 Stock (mL)
$Ge_{0.698}Si_{0.252}Sn_{0.050}$	1.560	2.64
$Ge_{0.745}Si_{0.209}Sn_{0.046}$	1.524	2.16
$Ge_{0.779}Si_{0.177}Sn_{0.044}$	1.500	1.80
$Ge_{0.785}Si_{0.158}Sn_{0.057}$	1.512	1.56
$Ge_{0.810}Si_{0.136}Sn_{0.054}$	1.476	1.44
$Ge_{0.832}Si_{0.116}Sn_{0.052}$	1.440	1.20
$Ge_{0.845}Si_{0.107}Sn_{0.048}$	1.440	1.20
$Ge_{0.855}Si_{0.087}Sn_{0.058}$	1.416	0.84
$Ge_{0.879}Si_{0.077}Sn_{0.044}$	1.392	0.84
$Ge_{0.888}Si_{0.066}Sn_{0.046}$	1.358	0.96
$Ge_{0.907}Si_{0.040}Sn_{0.053}$	1.356	0.60
$Ge_{0.911}Si_{0.030}Sn_{0.059}$	1.356	0.60

Table S2. Elemental composition of selected $Ge_{1-x-y}Si_ySn_x$ QDs obtained from EDS and XPS analyses.

Average Composition from EDS[a]	Average Composition from XPS[b]	Alloy Composition (in the core) based only on zero oxidation state species obtained from XPS[c]
$Ge_{0.745}Si_{0.209}Sn_{0.046}$	$Ge_{0.778}Si_{0.171}Sn_{0.059}$	$Ge_{0.934}Si_{0.038}Sn_{0.028}$
$Ge_{0.810}Si_{0.136}Sn_{0.054}$	$Ge_{0.777}Si_{0.141}Sn_{0.081}$	$Ge_{0.931}Si_{0.016}Sn_{0.053}$
$Ge_{0.863}Si_{0.082}Sn_{0.066}$	$Ge_{0.851}Si_{0.079}Sn_{0.070}$	$Ge_{0.958}Si_{0.008}Sn_{0.034}$
$Ge_{0.885}Si_{0.068}Sn_{0.047}$	$Ge_{0.861}Si_{0.077}Sn_{0.062}$	$Ge_{0.976}Si_{0.001}Sn_{0.023}$

[a] Average compositions were obtained from SEM-EDS analysis of 5 individual measurements per sample. [b]XPS compositions were obtained as the ratio of the integrated area under the deconvoluted peaks of respective chemical species, calculated using the sensitivity factors of 0.535, 0.368, and 4.89 for Ge 3d, Si 2p, and Sn 3d, respectively.[1] [c] The alloy compositions in the core were calculated using area percentiles of the deconvoluted, zero oxidation state peaks (Ge^0, Si^0, and Sn^0) shown in Figure 4 and Supporting Information, Figure S10. The corresponding composition analysis of uncharged species is provided in Table S4.

Table S3. Raman peak positions and average particle sizes of $Ge_{1-x-y}Si_ySn_x$ QDs, along with size dispersity from polydispersity index and the coefficient of variation.

QD Composition	Ge-Ge Raman (cm^{-1})	Ge-Si Raman (cm^{-1})	Mean Size (nm)[a]	Standard Deviation (nm)[a]	Polydispersity Index (PDI)[b]	Coefficient of Variation (CV%)
$Ge_{0.698}Si_{0.252}Sn_{0.050}$	290.1	436.6	4.1	0.3	0.0054	7.32
$Ge_{0.745}Si_{0.209}Sn_{0.046}$	290.6	435.6	4.3	0.5	0.0135	11.63
$Ge_{0.779}Si_{0.177}Sn_{0.044}$	289.5	435.3	4.0	0.4	0.0100	10.00
$Ge_{0.785}Si_{0.158}Sn_{0.057}$	289.3	434.1	4.7	0.5	0.0113	10.64
$Ge_{0.810}Si_{0.136}Sn_{0.054}$	289.4	432.1	4.2	0.6	0.0204	14.29
$Ge_{0.832}Si_{0.116}Sn_{0.052}$	290.1	433.6	5.1	0.6	0.0138	11.76
$Ge_{0.845}Si_{0.107}Sn_{0.048}$	289.3	432.6	4.8	0.7	0.0213	14.58
$Ge_{0.855}Si_{0.087}Sn_{0.058}$	290.1	432.6	4.9	0.7	0.0204	14.29
$Ge_{0.879}Si_{0.077}Sn_{0.044}$	290.1	431.1	4.5	0.6	0.0178	13.33
$Ge_{0.888}Si_{0.066}Sn_{0.046}$	289.5	N/A	4.4	0.5	0.0129	11.36
$Ge_{0.907}Si_{0.040}Sn_{0.053}$	288.9	N/A	4.7	0.6	0.0163	12.77
$Ge_{0.911}Si_{0.030}Sn_{0.059}$	288.9	N/A	5.2	0.6	0.0133	11.54

[a] Mean sizes and corresponding standard deviations were obtained from TEM analysis of 200-300 particles per composition.

[a]PDI is dimensionless and used as a measure of the degree of non-uniformity of a sample of particles. Values were obtained as the squared relative standard deviation of the particle size distribution as defined in the ISO standard documents 13321:1996 E and ISO 22412:2008.[2,3] It is scaled such that values smaller than 0.05 are described as highly monodisperse, and values larger than 0.7 indicate that the sample has a broad particle size distribution.

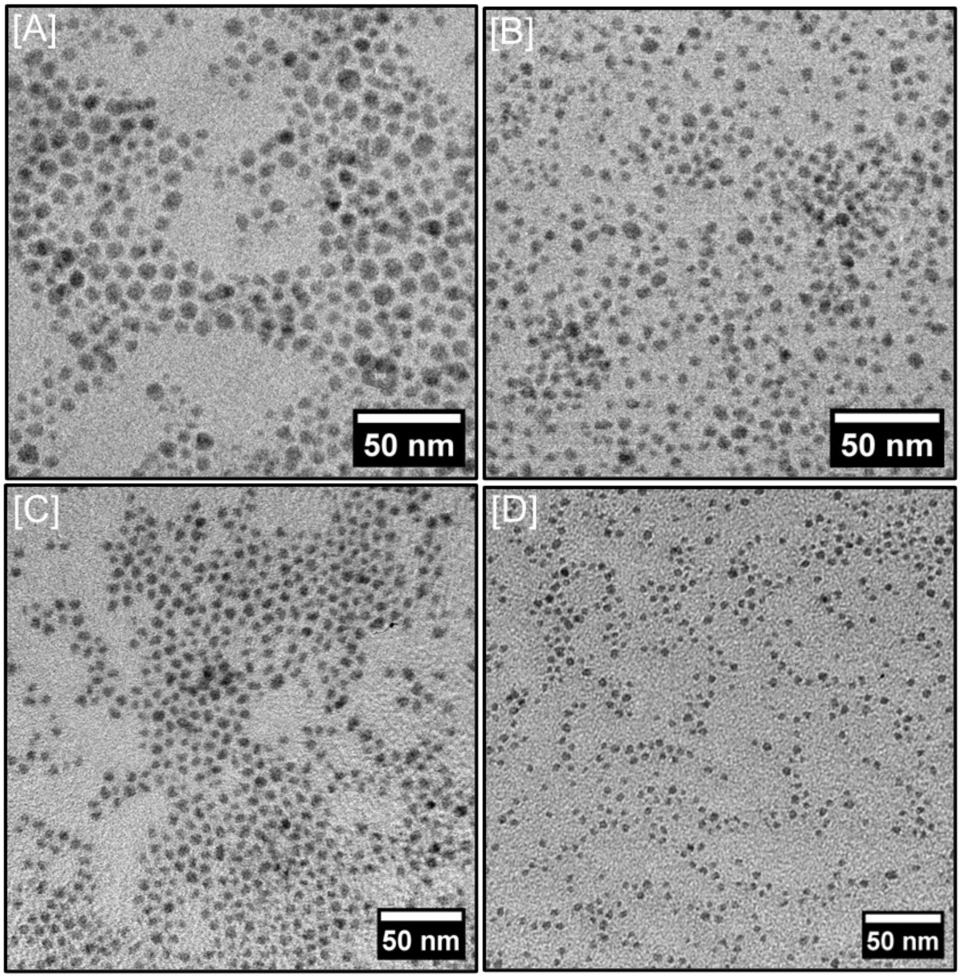

Figure S1. LRTEM images of $Ge_{0.830}Si_{0.120}Sn_{0.050}$ alloy QD fractions obtained from size selective precipitation. The fractionation process yielded four distinct sizes: [A] Fraction 1 (6.1 ± 1.5 nm), [B] Fraction 2 (5.7 ± 0.9 nm), [C]. Fraction 3 (4.8 ± 0.7 nm), and [D] Fraction 4 (4.4 ± 0.5 nm). The average particle sizes are shown in parentheses.

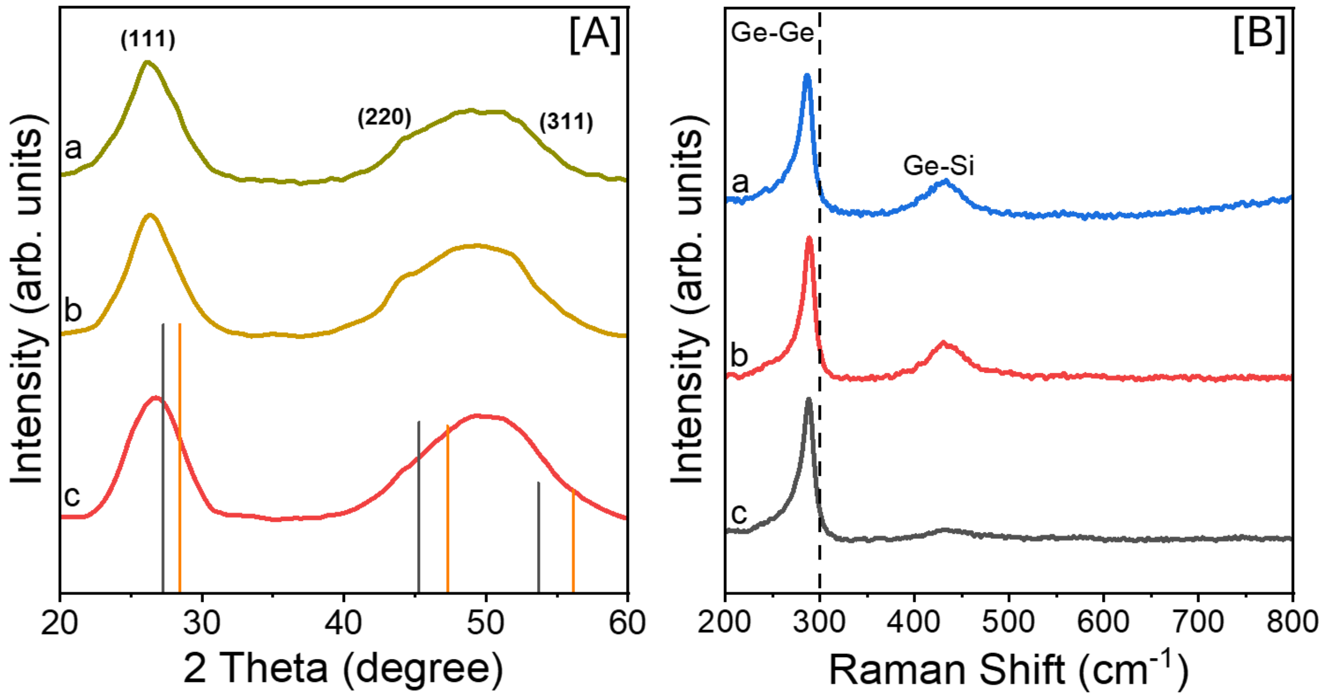

Figure S2. [A] PXRD patterns and [B] Raman spectra of ternary alloy QDs with variable compositions: (a) $Ge_{0.785}Si_{0.158}Sn_{0.057}$, (b) $Ge_{0.845}Si_{0.107}Sn_{0.048}$, and (c) $Ge_{0.907}Si_{0.040}Sn_{0.053}$. The ICCD-PDF overlay of diamond cubic Ge (ICCD # 04-002-0892) and Si (ICCD # 01-085-8586) are shown in vertical black and orange lines, respectively. The dashed line in [B] represents the bulk Ge−Ge vibration at 300 cm^{-1}.

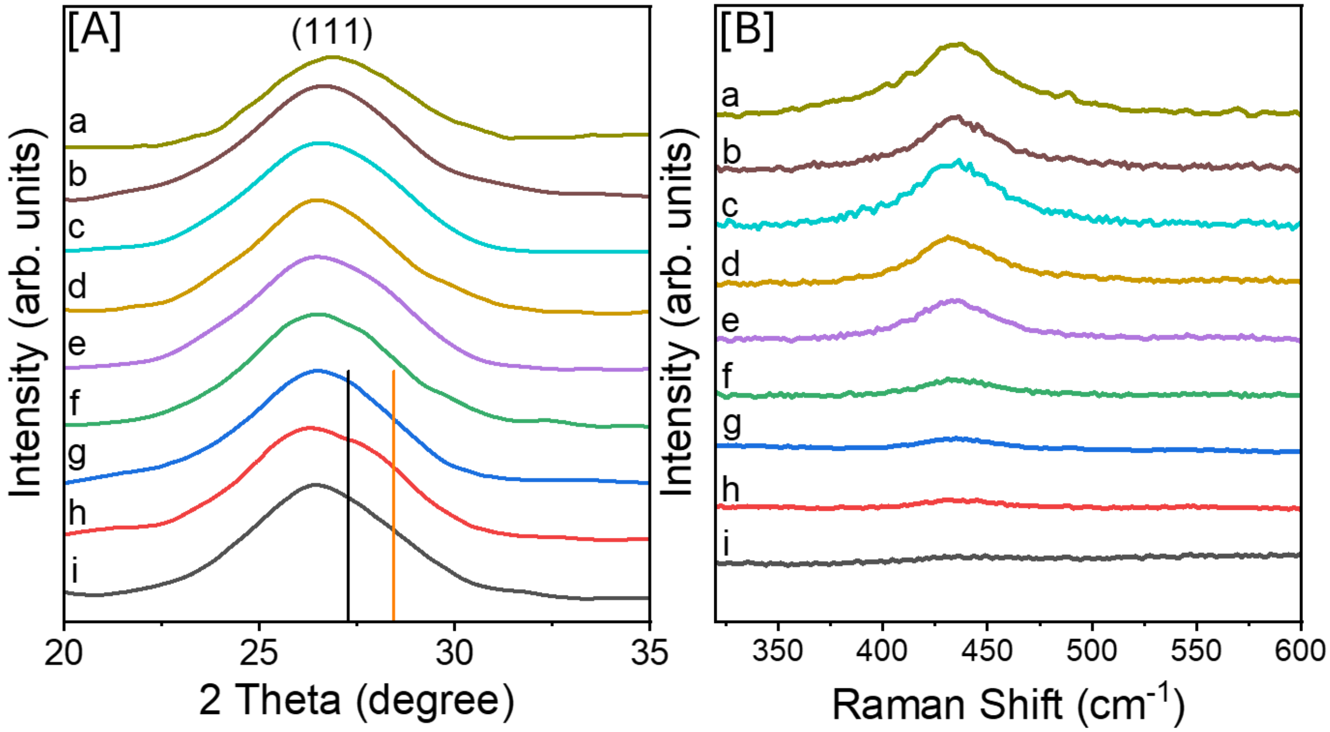

Figure S3. [A] PXRD and [B] Raman spectra of alloy QDs with variable Si and Sn compositions: (a) $Ge_{0.698}Si_{0.252}Sn_{0.050}$, (b) $Ge_{0.745}Si_{0.209}Sn_{0.046}$, (c) $Ge_{0.779}Si_{0.177}Sn_{0.044}$, (d) $Ge_{0.810}Si_{0.136}Sn_{0.054}$, (e) $Ge_{0.832}Si_{0.116}Sn_{0.052}$, (f) $Ge_{0.855}Si_{0.087}Sn_{0.058}$, (g) $Ge_{0.879}Si_{0.077}Sn_{0.044}$, (h) $Ge_{0.888}Si_{0.066}Sn_{0.046}$, and (i) $Ge_{0.911}Si_{0.030}Sn_{0.059}$. PXRD patterns were zoomed in to display (111) reflection of diamond cubic Ge. The ICCD-PDF overlays of diamond cubic Ge (ICCD # 04-002-0892) and Si (ICCD # 01-085-8586) are shown as vertical black and orange lines, respectively. Raman spectra were zoomed in to show Ge-Si phonon mode at ~431 – 437 cm^{-1}.

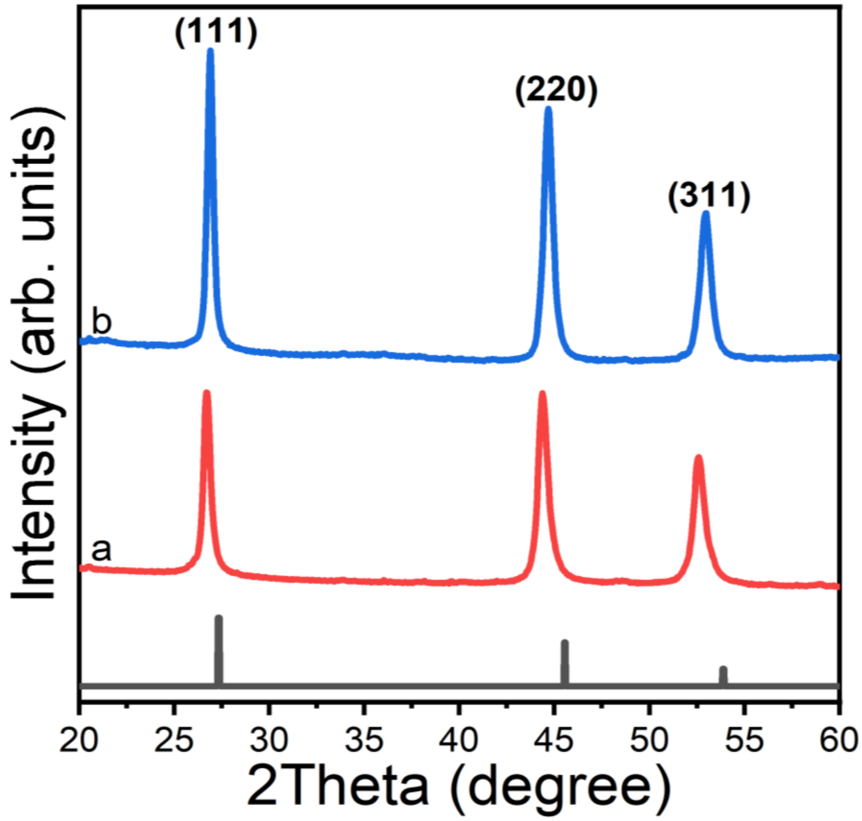

Figure S4. PXRD patterns of bulk-like $Ge_{0.867}Si_{0.080}Sn_{0.053}$ particles with crystallite sizes of (a) 18.1 nm and (b) 22.7 nm. The ICCD-PDF overlay of diamond cubic Ge (ICCD # 04-002-0892) is shown as a vertical black line.

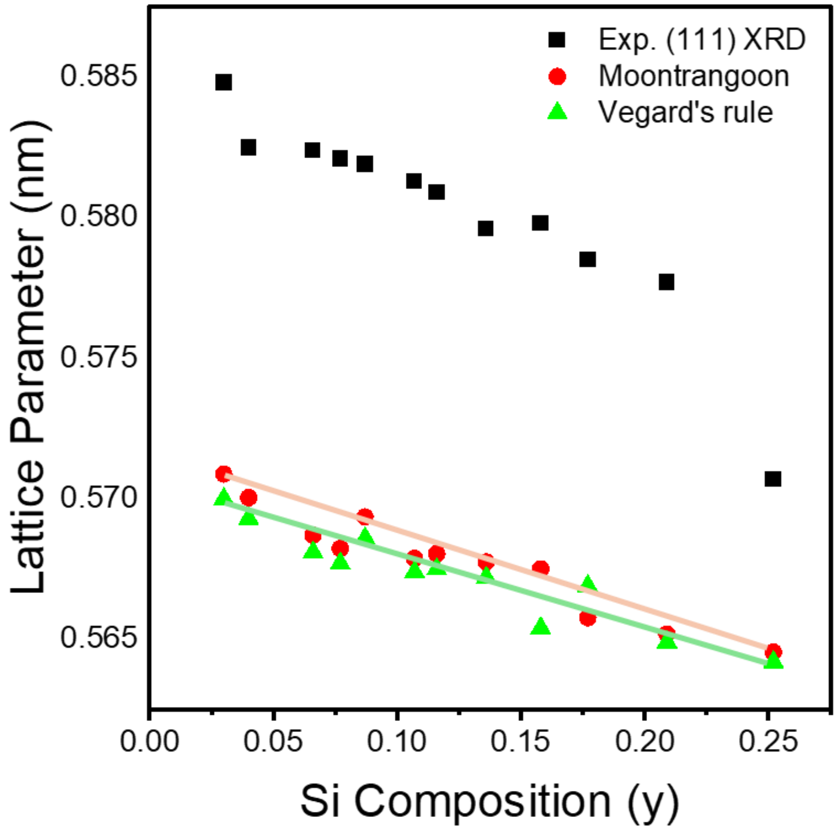

Figure S5. Lattice parameter variation with Si composition of $Ge_{1-x-y}Si_ySn_x$ alloy QDs. Experimental lattice parameters were computed using (111) diffraction peaks of PXRD patterns, following pseudo-Voigt fits. The experimental Si compositions were obtained from SEM/EDS analysis. Theoretical lattice parameters were calculated based on Moontragoon's method[4] and Vegard's rule. [5,6]

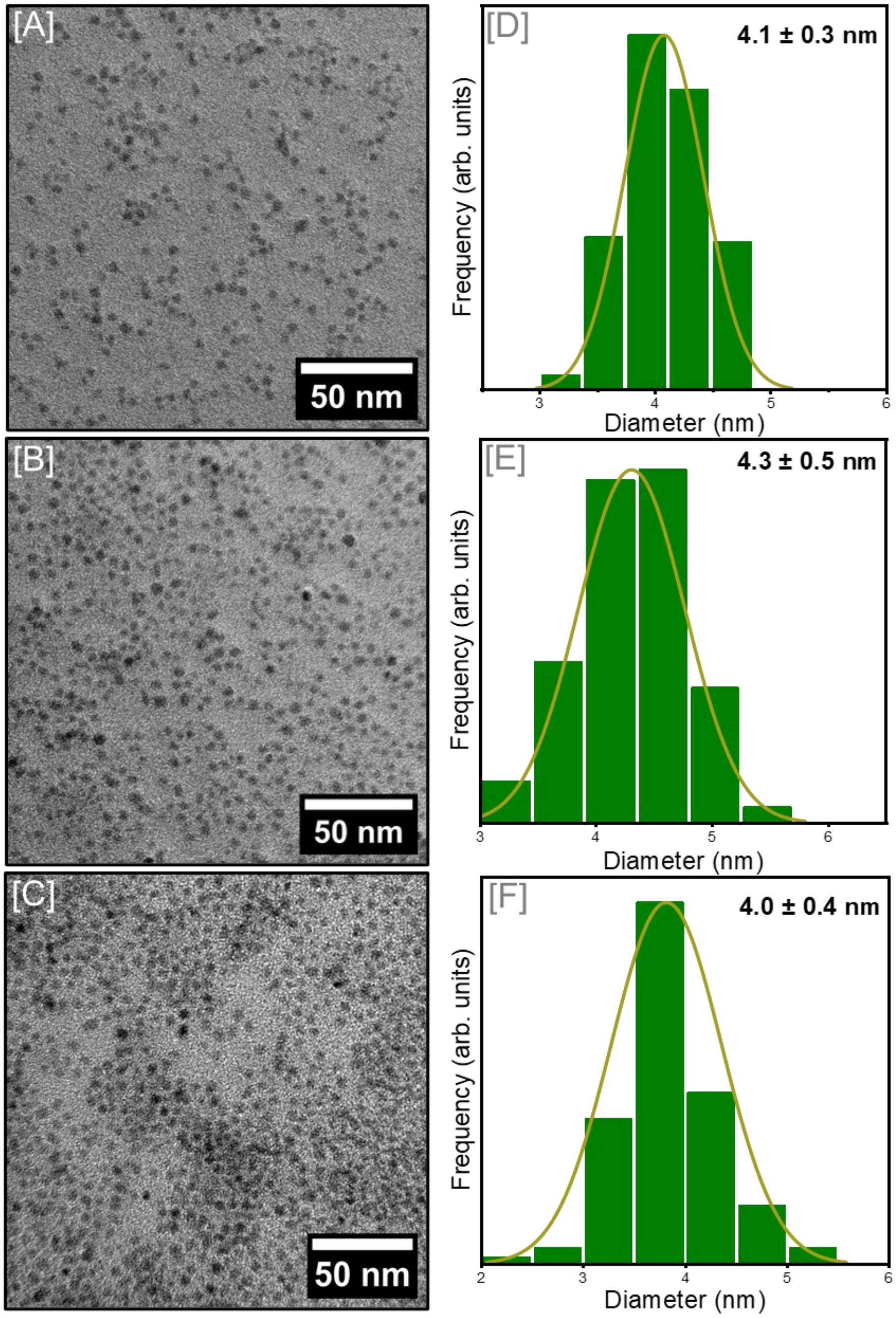

Figure S6. Representative LRTEM images and corresponding size histograms of $Ge_{1-x-y}Si_ySn_x$ alloy QDs: [A, D] $Ge_{0.698}Si_{0.252}Sn_{0.050}$, [B, E] $Ge_{0.745}Si_{0.209}Sn_{0.046}$, and [C, F] $Ge_{0.779}Si_{0.177}Sn_{0.044}$.

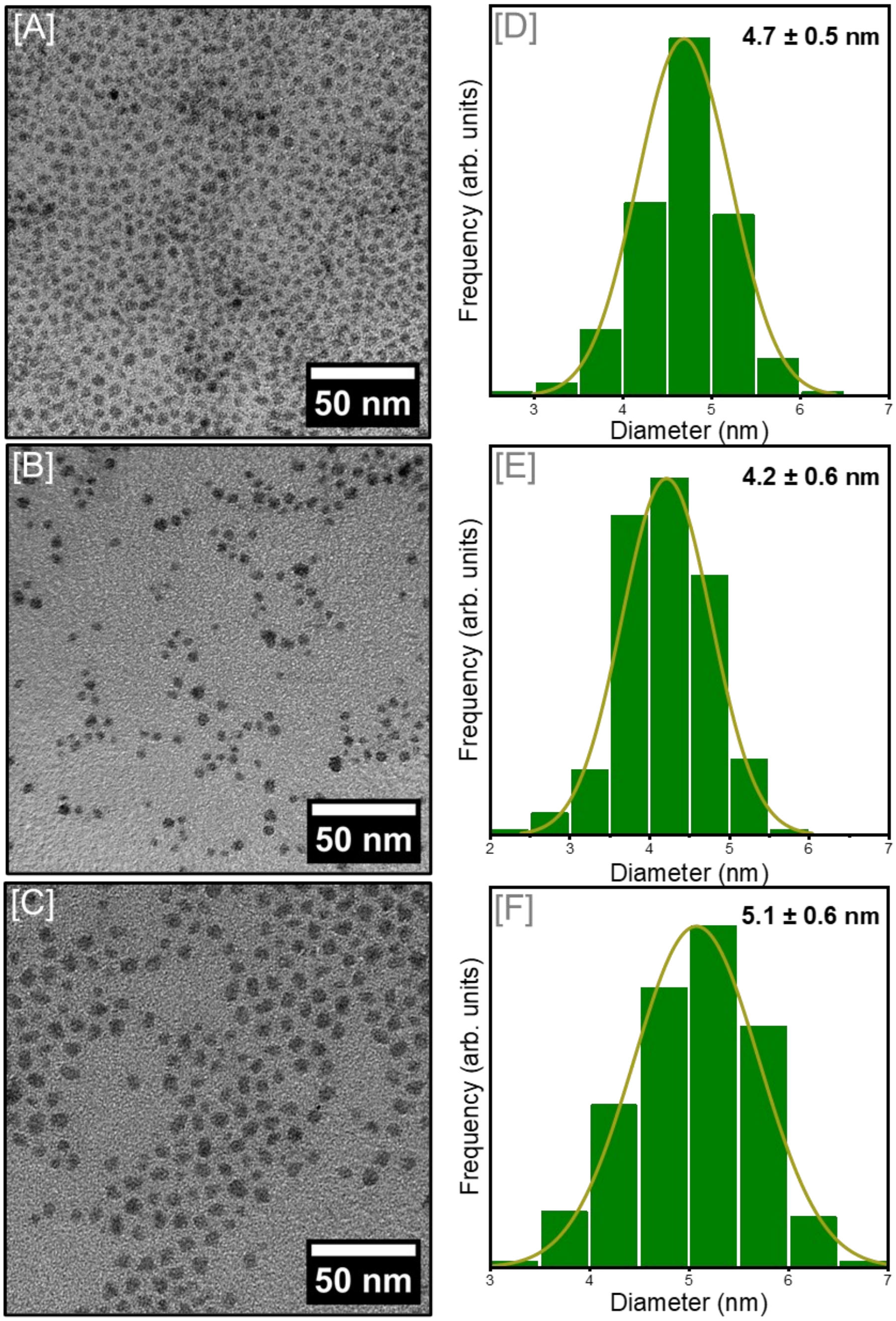

Figure S7. Representative LRTEM images and corresponding size histograms of $Ge_{1-x-y}Si_ySn_x$ alloy QDs: [A, D] $Ge_{0.785}Si_{0.158}Sn_{0.057}$, [B, E] $Ge_{0.810}Si_{0.136}Sn_{0.054}$, and [C, F] $Ge_{0.832}Si_{0.116}Sn_{0.052}$.

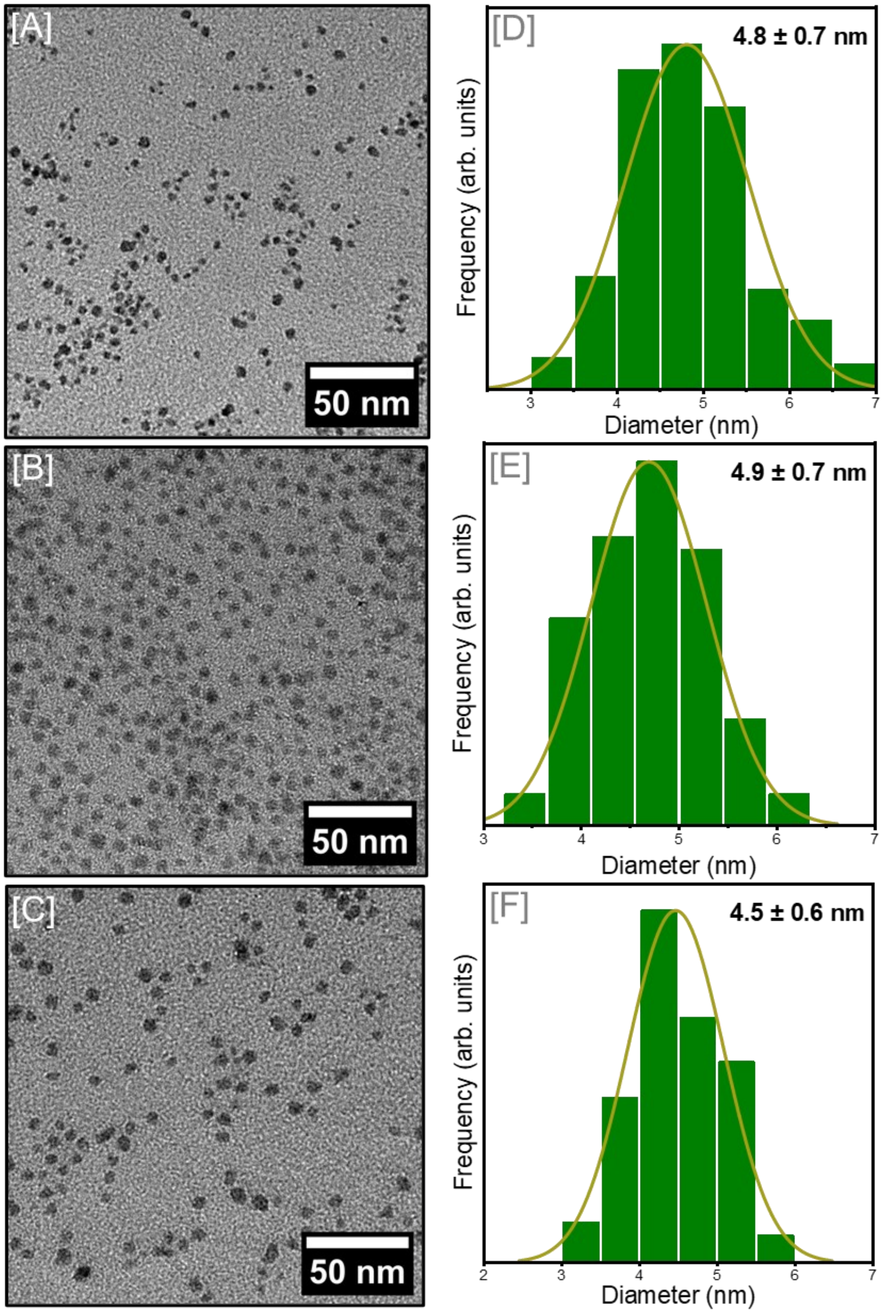

Figure S8. Representative LRTEM images and corresponding size histograms of $Ge_{1-x-y}Si_ySn_x$ alloy QDs: [A, D] $Ge_{0.845}Si_{0.107}Sn_{0.048}$, [B, E] $Ge_{0.855}Si_{0.087}Sn_{0.058}$, and [C, F] $Ge_{0.879}Si_{0.077}Sn_{0.044}$.

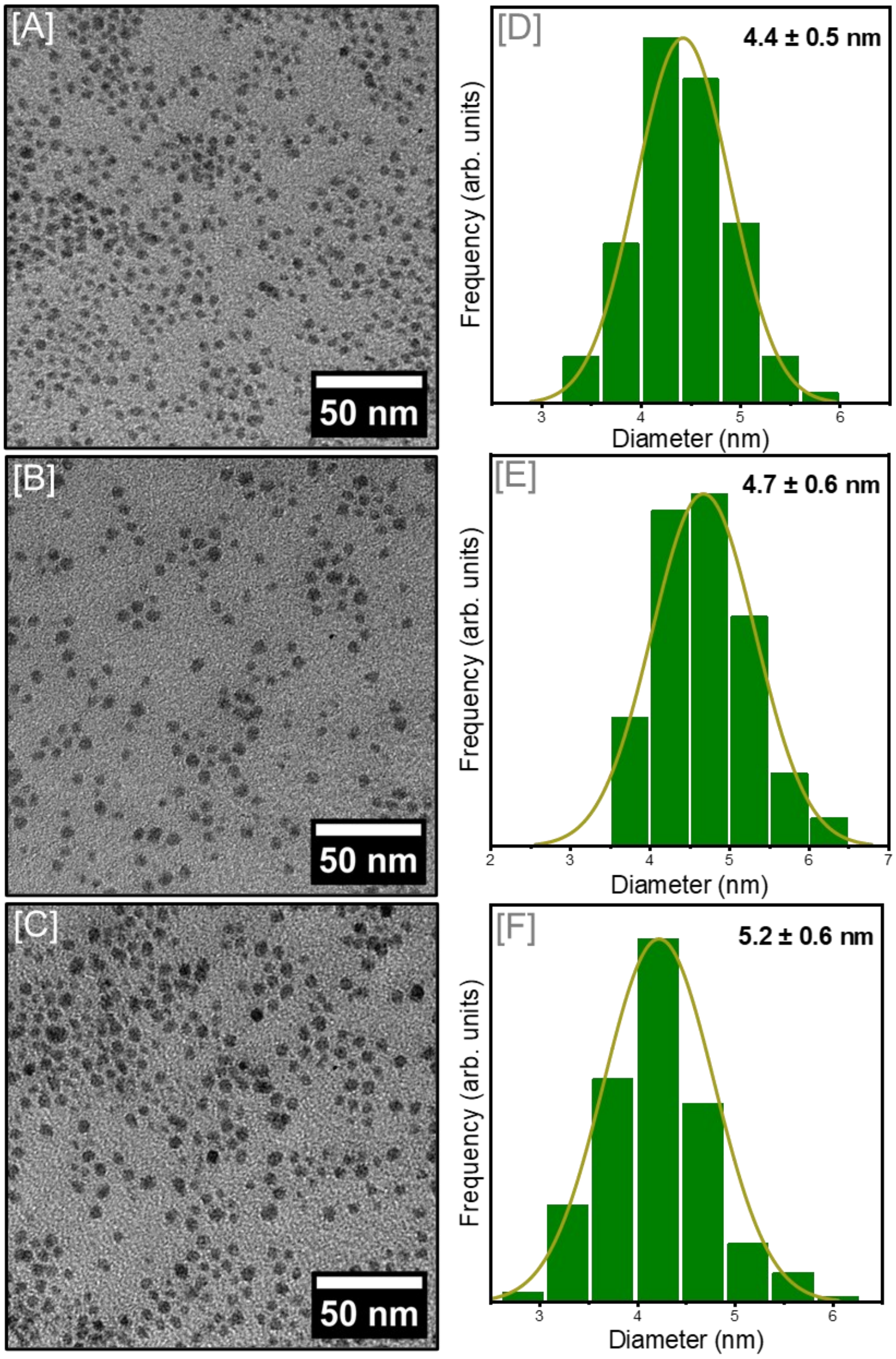

Figure S9. Representative LRTEM images and corresponding size histograms of $Ge_{1-x-y}Si_ySn_x$ alloy QDs: [A, D] $Ge_{0.888}Si_{0.066}Sn_{0.046}$, [B, E] $Ge_{0.907}Si_{0.040}Sn_{0.053}$, and [C, F] $Ge_{0.911}Si_{0.030}Sn_{0.059}$.

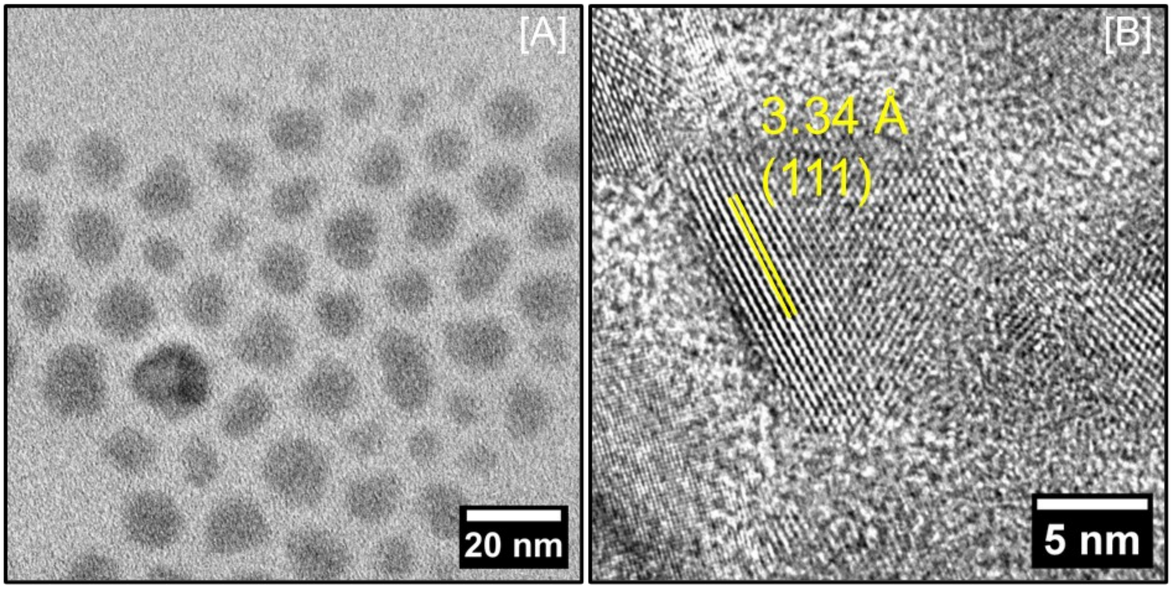

Figure S10. [A] LRTEM and [B] HRTEM images of larger (8.2 ± 1.5 nm) $Ge_{0.84}Si_{0.11}Sn_{0.05}$ alloy nanocrystals. Figure B shows lattice fringes corresponding to an expanded (111) plane of diamond cubic Ge.

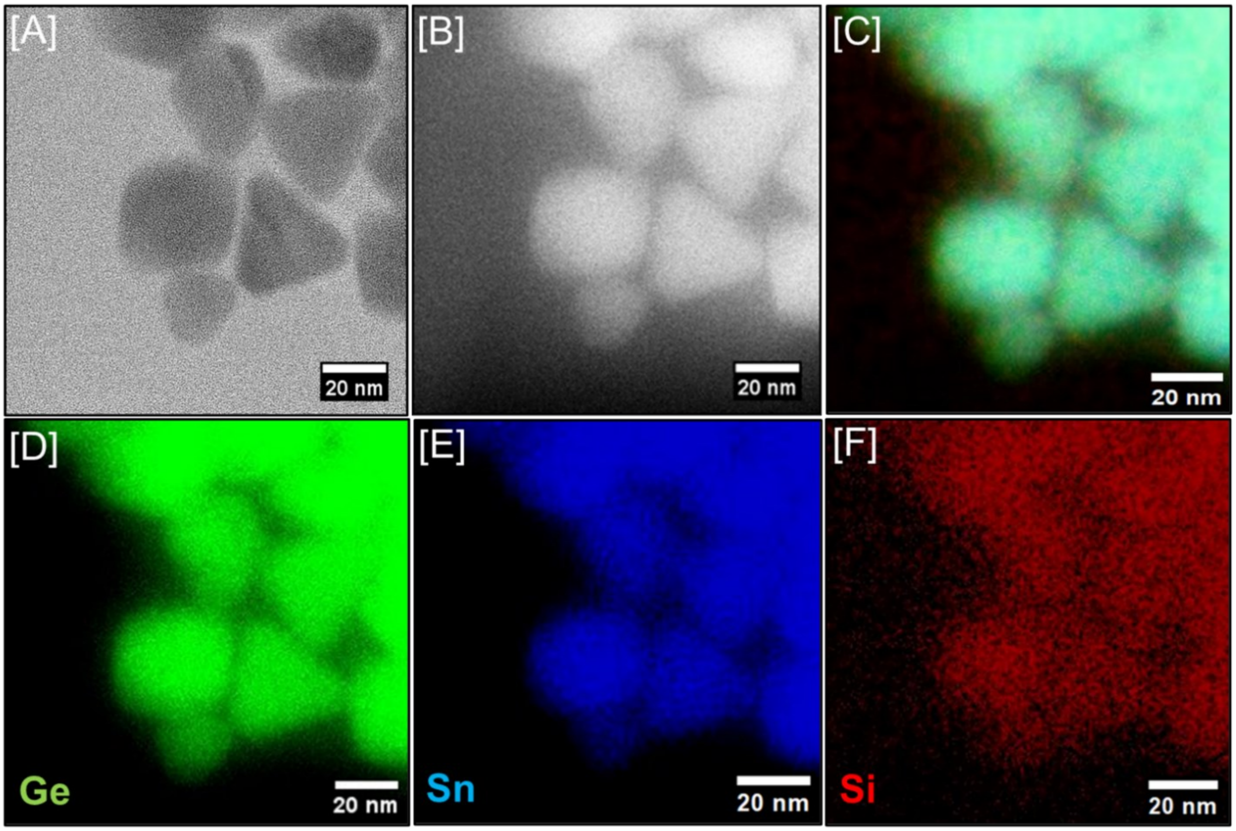

Figure S11. [A] Bright field and [B] Dark-field TEM images of $Ge_{0.68}Si_{0.16}Sn_{0.16}$ alloys along with STEM-HAADF elemental maps of [D] Ge, [E] Sn, [F] Si, and [C] an overlay of all elements, confirming the homogeneous solid solution behavior of as-synthesized alloys.

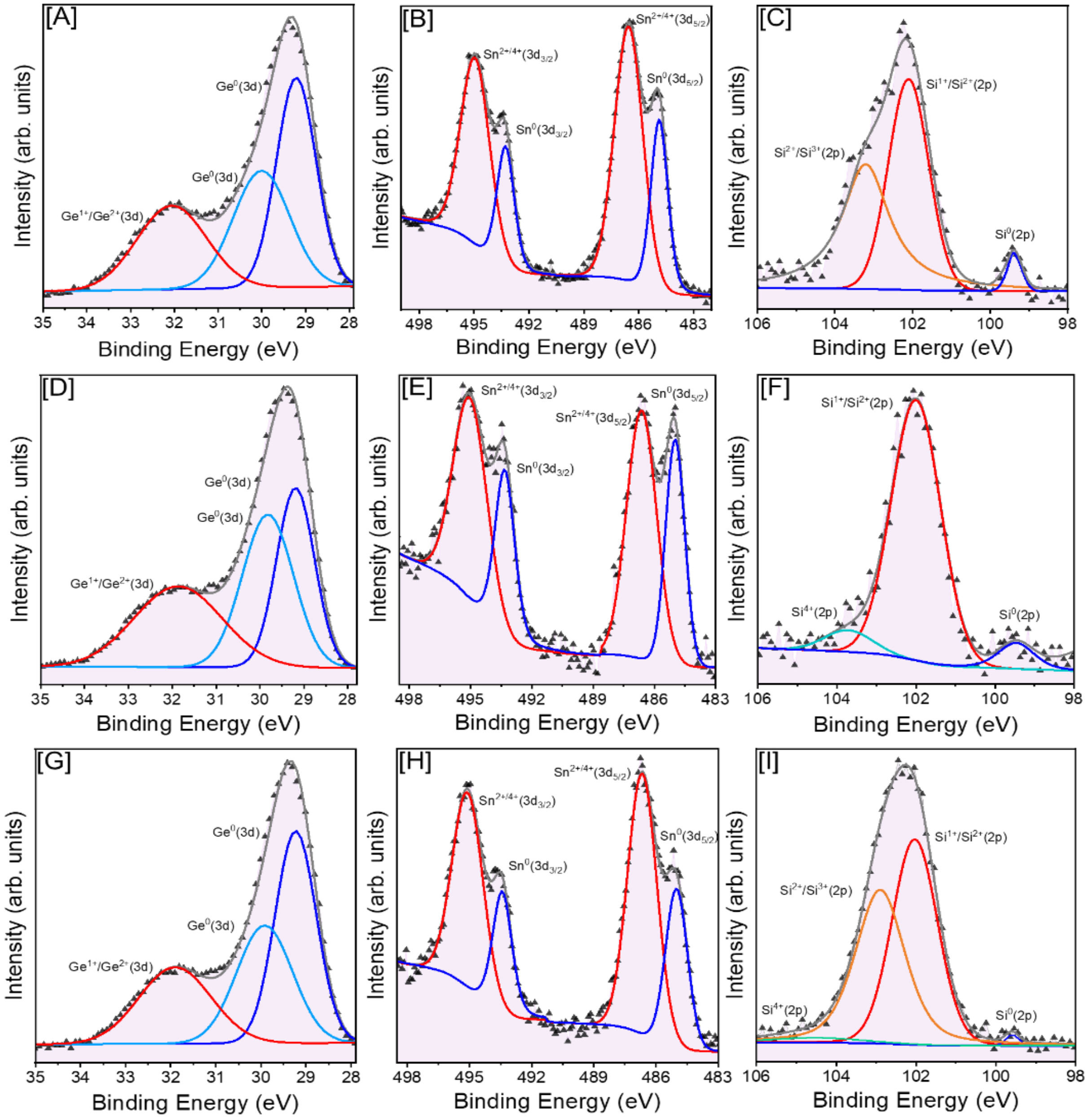

Figure S12. Ge 3d, Sn 3d, and Si 2p XPS spectra of $Ge_{1-x-y}Si_ySn_x$ alloy QDs: [A, B, C] $Ge_{0.745}Si_{0.209}Sn_{0.046}$, [D, E, F] $Ge_{0.863}Si_{0.082}Sn_{0.066}$, and [G, H, I] $Ge_{0.885}Si_{0.068}Sn_{0.047}$. The black symbols represent the experimental data, blue colored lines are fitted deconvolution peaks for Ge^0/Si^0/Sn^0 species, red, green, and orange lines are fitted deconvolutions for charged surface species, and the gray colored lines represent spectral envelopes.

Table S4. XPS integrated area under the deconvoluted peaks for Ge 3d, Sn 3d, and Si 2p regions of selected QD samples, highlighting the relative contributions from core species.

Average Composition from EDS	% Area		
	Ge^0	Si^0	Sn^0
$Ge_{0.745}Si_{0.209}Sn_{0.046}$	74.3	10.8	36.0
$Ge_{0.810}Si_{0.136}Sn_{0.054}$	75.4	7.7	64.7
$Ge_{0.863}Si_{0.082}Sn_{0.066}$	74.8	6.5	34.4
$Ge_{0.885}Si_{0.068}Sn_{0.047}$	76.8	1.3	33.6

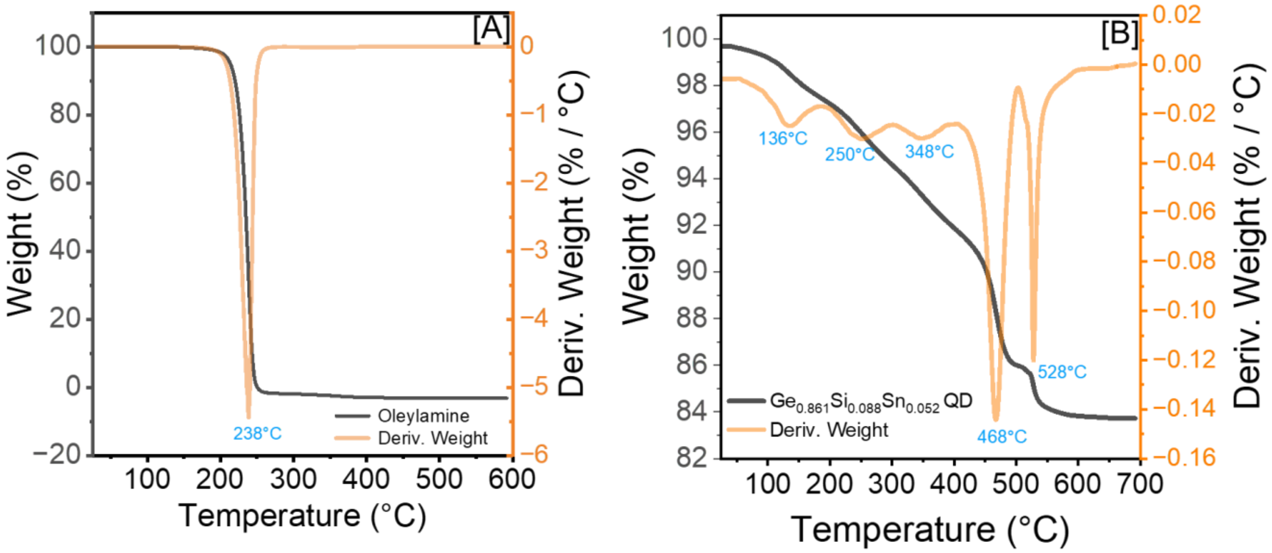

Figure S13. TGA curves of [A] free OLA and [B] OLA passivated $Ge_{0.861}Si_{0.088}Sn_{0.052}$ QDs synthesized at 300 °C. The first derivatives of corresponding TGA plots are overlaid to show major weight losses.

TGA measurements were performed under inert conditions to probe the efficacy of surface passivation. TGA curve of free OLA (Figure S13A) shows a single step and sharp weight loss at 238 °C (peak maxima of the first derivative) that can be attributed to decomposition of OLA, consistent with literature reports.[7,8] For the OLA passivated $Ge_{0.861}Si_{0.088}Sn_{0.052}$ QDs (Figure S13B), a broad mass loss curve was observed extending the ligand decomposition to ~530 °C. The apex of the two major weight loss events occurred at 468 °C and 528 °C, suggesting strong QD-ligand interactions. The initial broad weight loss below 400 °C can be attributed to loss of trapped moisture (≤ 136 °C) and desorption of weakly adsorbed OLA, ODE, and/or butyl surface ligands (250 °C extending up to 348 °C).[9] The weight losses at 468 °C and 528 °C indicate the desorption and complete burning off the passivating surface ligands. A residual material was collected post-

TGA analysis (~700 °C) for QDs, while no stable residue was obtained for the free OLA sample. The observed decomposition temperature of OLA-coated QDs is consistent with literature reports on alkylamine passivated nanostructures.[7,9,10]

Table S5. Energy gaps in eV derived via Kubelka Munk absorption onsets, Tauc direct and indirect-gap equations, and Boltzmann direct-gap (n = 0.9) analysis[11] of $Ge_{1-x-y}Si_ySn_x$ alloy QDs.

QD Composition	Absorption Onset	Tauc Indirect (E_g^{Ind})	Tauc Direct (E_g^{Dir})	Boltzmann Direct
$Ge_{0.698}Si_{0.252}Sn_{0.050}$	2.33	1.81	3.03	2.84
$Ge_{0.745}Si_{0.209}Sn_{0.046}$	2.09	1.62	2.54	2.61
$Ge_{0.779}Si_{0.177}Sn_{0.044}$	1.97	1.59	2.43	2.43
$Ge_{0.785}Si_{0.158}Sn_{0.057}$	1.73	1.49	2.32	2.31
$Ge_{0.810}Si_{0.136}Sn_{0.054}$	1.53	1.43	2.17	2.12
$Ge_{0.845}Si_{0.107}Sn_{0.048}$	1.50	1.43	1.93	2.09
$Ge_{0.855}Si_{0.087}Sn_{0.058}$	1.30	1.08	2.02	1.92
$Ge_{0.888}Si_{0.066}Sn_{0.046}$	1.30	1.19	1.91	1.79
$Ge_{0.911}Si_{0.030}Sn_{0.059}$	1.15	1.01	1.85	1.46

Table S6. Emission parameters of $Ge_{1-x-y}Si_ySn_x$ alloy QDs from which PL was observed.

QD Composition	PL Peak Energy (eV)	PL FWHM (eV)
$Ge_{0.698}Si_{0.252}Sn_{0.050}$	2.42	0.68
$Ge_{0.710}Si_{0.237}Sn_{0.053}$	2.38	0.81
$Ge_{0.745}Si_{0.209}Sn_{0.046}$	2.41	0.63
$Ge_{0.779}Si_{0.177}Sn_{0.044}$	2.32	0.95
$Ge_{0.814}Si_{0.134}Sn_{0.052}$	1.85	0.46
$Ge_{0.825}Si_{0.121}Sn_{0.054}$	1.84	0.47
$Ge_{0.856}Si_{0.094}Sn_{0.050}$	1.86	0.47
$Ge_{0.872}Si_{0.082}Sn_{0.046}$	1.86	0.49
$Ge_{0.872}Si_{0.076}Sn_{0.053}$	1.86	0.47
$Ge_{0.885}Si_{0.068}Sn_{0.047}$	1.87	0.48

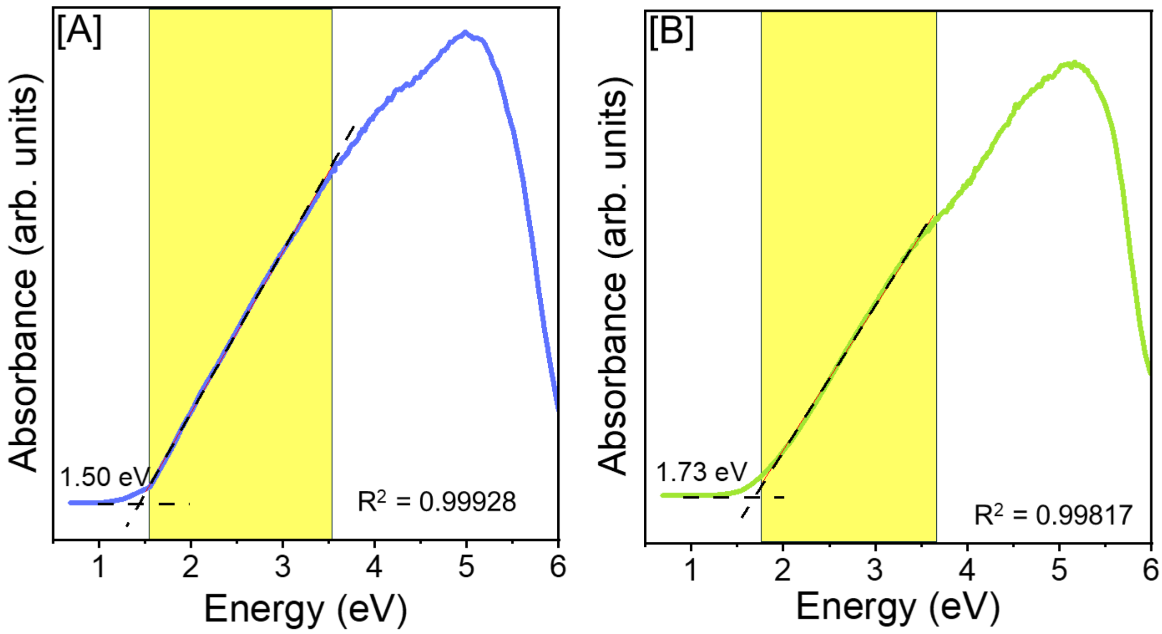

Figure S14: Solid-state absorption spectra of [A] $Ge_{0.832}Si_{0.116}Sn_{0.052}$ and [B] $Ge_{0.785}Si_{0.158}Sn_{0.057}$ alloy QDs illustrating fits used in analysis of absorption onsets with corresponding R^2 values shown. A similar study was performed on other QD compositions to determine corresponding absorption onsets.

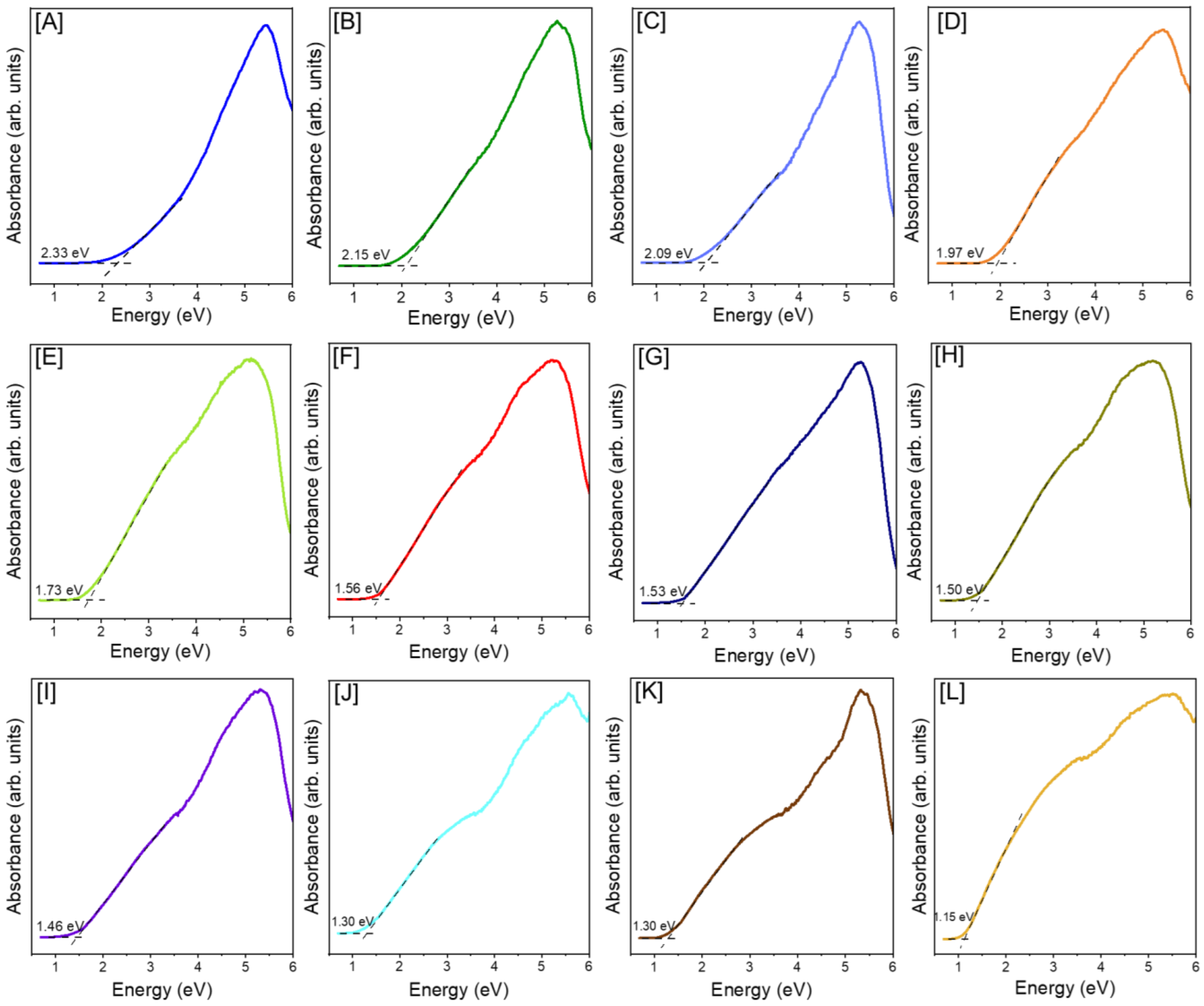

Figure S15. Solid-state absorption spectra of $Ge_{1-x-y}Si_ySn_x$ alloy QDs with variable Si composition: [A] $Ge_{0.698}Si_{0.252}Sn_{0.050}$, [B] $Ge_{0.710}Si_{0.237}Sn_{0.053}$, [C] $Ge_{0.745}Si_{0.209}Sn_{0.046}$, [D] $Ge_{0.779}Si_{0.177}Sn_{0.044}$, [E] $Ge_{0.785}Si_{0.158}Sn_{0.057}$, [F] $Ge_{0.814}Si_{0.134}Sn_{0.052}$, [G] $Ge_{0.825}Si_{0.121}Sn_{0.054}$, [H] $Ge_{0.845}Si_{0.107}Sn_{0.048}$, [I] $Ge_{0.856}Si_{0.094}Sn_{0.050}$, [J] $Ge_{0.872}Si_{0.082}Sn_{0.046}$, [K] $Ge_{0.885}Si_{0.068}Sn_{0.047}$, [L] $Ge_{0.911}Si_{0.030}Sn_{0.059}$. Absorption onsets were determined using Kubelka-Munk analysis.

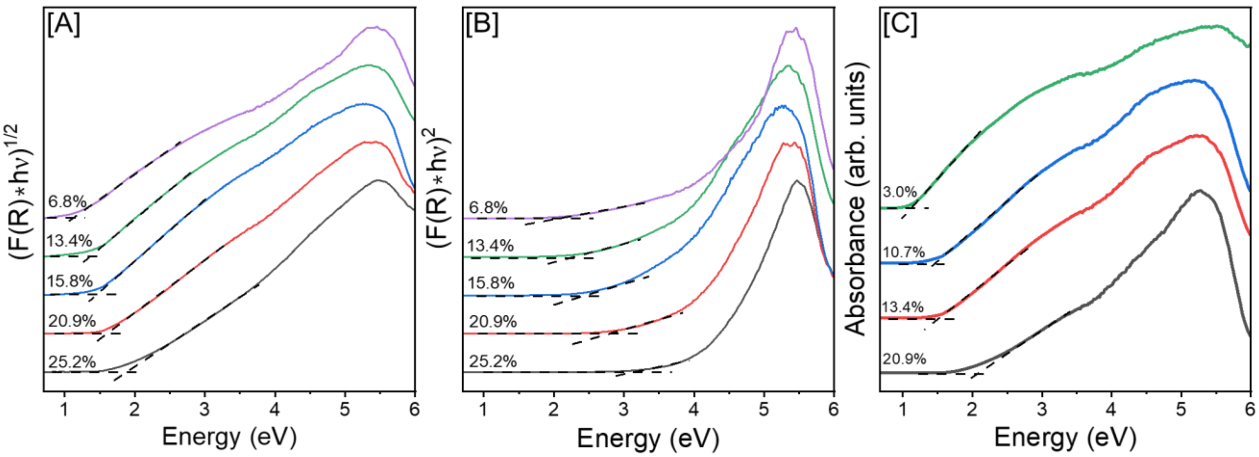

Figure S16. Representative [A] Tauc indirect-gap, [B] Tauc direct-gap, and [C] additional solid-state absorption spectra of $Ge_{1-x-y}Si_ySn_x$ alloy QDs. The corresponding Si composition obtained from the SEM-EDS analysis is shown with each spectrum.

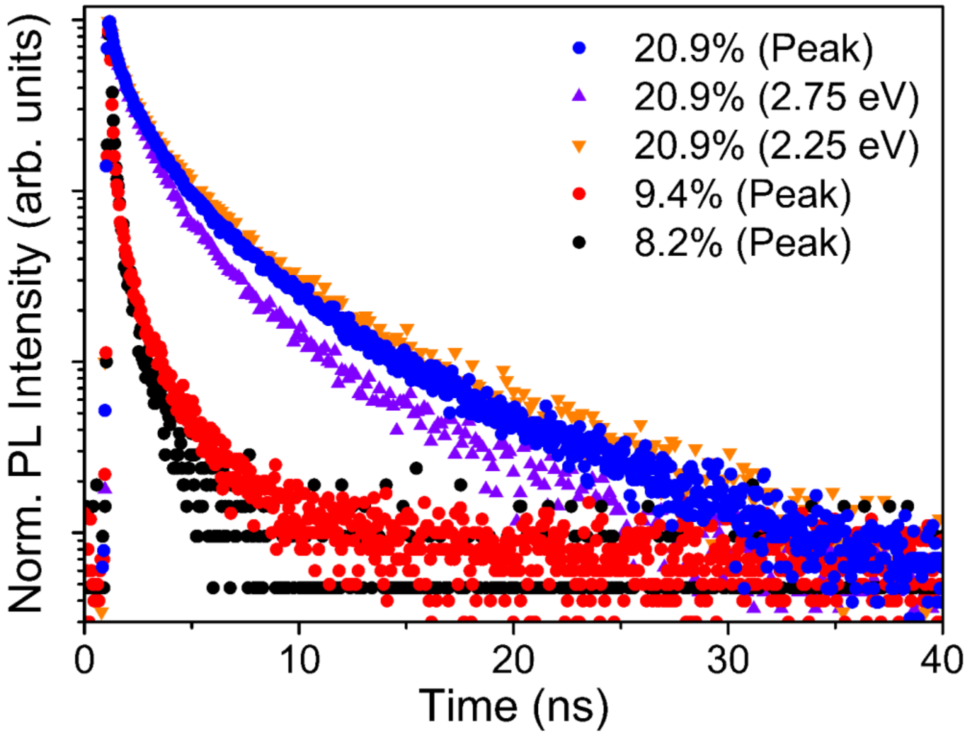

Figure S17. Room-temperature time-resolved PL decays obtained for a ~75 meV bandwidth around emission peaks of $Ge_{0.745}Si_{0.209}Sn_{0.046}$, $Ge_{0.856}Si_{0.094}Sn_{0.050}$, and $Ge_{0.872}Si_{0.082}Sn_{0.046}$ alloy QDs. Average fitted time constants notably increased with Si incorporation, being 0.41 ns (y = 0.082), 0.58 ns (y = 0.094), and 3.39 ns (y = 0.209). Purple upwards and orange downwards triangles denote decays measured above (2.75 eV, τ_{Av} = 2.59 ns) and below (2.25 eV, τ_{Av} = 3.56 ns) the emission peak of the highest Si composition sample ($Ge_{0.745}Si_{0.209}Sn_{0.046}$) and are shown as vertical lines in Figure S18.

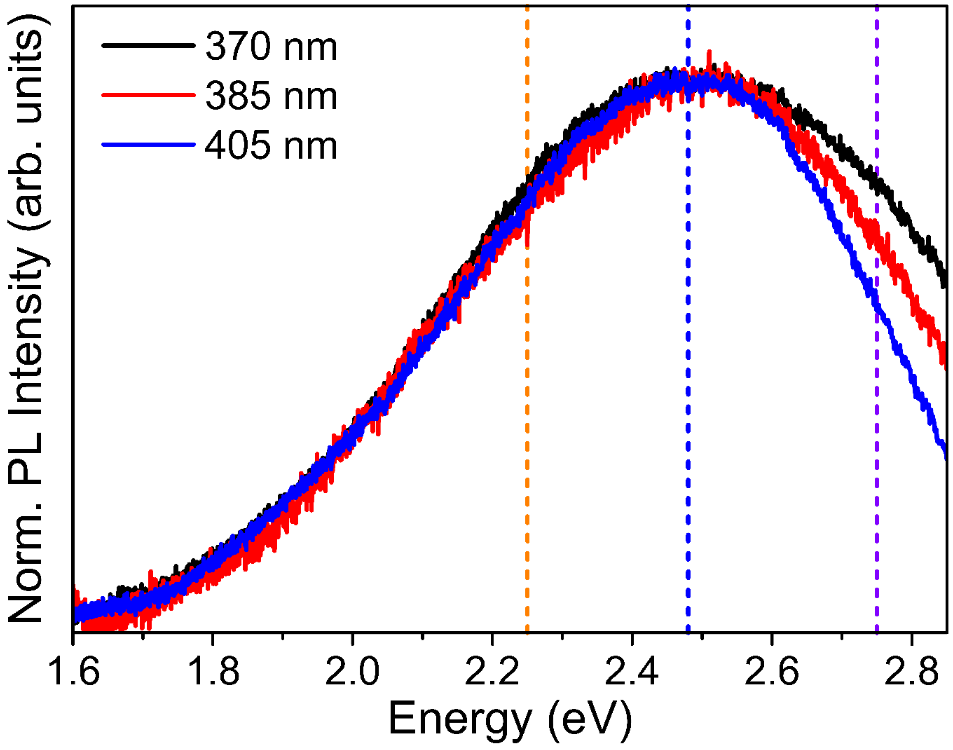

Figure S18. Normalized PL spectra obtained from the $Ge_{0.745}Si_{0.209}Sn_{0.046}$ alloy QDs when excited at varying wavelengths between 370 – 405 nm. The spectral regions measured in Figure S17 are illustrated by vertical lines.

REFERENCES

(1) Jensen, D. S.; Kanyal, S. S.; Madaan, N.; Vail, M. A.; Dadson, A. E.; Engelhard, M. H.; Linford, M. R. Silicon (100)/SiO_2 by XPS. *Surf. Sci. Spectra* **2013**, *20*, 36–42.

(2) Danaei, M.; Dehghankhold, M.; Ataei, S.; Hasanzadeh Davarani, F.; Javanmard, R.; Dokhani, A.; Khorasani, S.; Mozafari, M. R. Impact of Particle Size and Polydispersity Index on the Clinical Applications of Lipidic Nanocarrier Systems. *Pharmaceutics* **2018**. *10*, 57.

(3) Clayton, K. N.; Salameh, J. W.; Wereley, S. T.; Kinzer-Ursem, T. L. Physical Characterization of Nanoparticle Size and Surface Modification Using Particle Scattering Diffusometry. *Biomicrofluidics* **2016**, *10*, 054107.

(4) Moontragoon, P.; Soref, R. A.; Ikonic, Z. The Direct and Indirect Bandgaps of Unstrained $Si_xGe_{1-x-y}Sn_y$ and Their Photonic Device Applications. *J. Appl. Phys*. **2012**, *112*, 073106.

(5) Dismukes, J. P.; Ekstrom, L.; Paff, R. J. Lattice Parameter and Density in Germanium-Silicon Alloys. *J. Phys. Chem*. **1964**, *68*, 3021–3027.

(6) Murphy, S. T.; Chroneos, A.; Jiang, C.; Schwingenschlögl, U.; Grimes, R. W. Deviations from Vegard's Law in Ternary III-V Alloys. *Phys. Rev. B* **2010**, *82*, 073201.

(7) Lan, F.; Bai, J.; Wang, H. The Preparation of Oleylamine Modified Micro-Size Sphere Silver Particles and Its Application in Crystalline Silicon Solar Cells. *RSC Adv*. **2018**, *8*, 16866–16872.

(8) Sperry, B. M.; Luscombe, C. K. Ligand Pyrolysis during Air-Free Inorganic Nanocrystal Synthesis. *Chem. Mater*. **2021**, *33*, 136–145.

(9) Lenin, R.; Joy, P. A. Role of Primary and Secondary Surfactant Layers on the Thermal Conductivity of Lauric Acid Coated Magnetite Nanofluids. *J. Phys. Chem. C* **2016**, *120*, 11640–11651.

(10) Sperry, B. M.; Kukhta, N. A.; Huang, Y.; Luscombe, C. K. Ligand Decomposition during Nanoparticle Synthesis: Influence of Ligand Structure and Precursor Selection. *Chem. Mater*. **2023**, *35*, 570–583.

(11) Zanatta, A. R. Revisiting the Optical Bandgap of Semiconductors and the Proposal of a Unified Methodology to Its Determination. *Sci. Rep*. **2019**, *9*, 11225.
